# Social network analysis of obsidian artefacts and Māori interaction in northern Aotearoa New Zealand

**DOI:** 10.1371/journal.pone.0212941

**Published:** 2019-03-14

**Authors:** Thegn N. Ladefoged, Caleb Gemmell, Mark McCoy, Alex Jorgensen, Hayley Glover, Christopher Stevenson, Dion O’Neale

**Affiliations:** 1 Anthropology, University of Auckland, Auckland, New Zealand; 2 Te Pūnaha Matatini, Auckland, New Zealand; 3 Physics, University of Auckland, Auckland, New Zealand; 4 Anthropology, Southern Methodist University, Dallas, Texas, United States of America; 5 Anthropology, Virginia Commonwealth University, Richmond, Virginia, United States of America; University at Buffalo - The State University of New York, UNITED STATES

## Abstract

Over the span of some 700 years the colonizing populations of Aotearoa New Zealand grew, with subsequent changes in levels of interaction and social affiliation. Historical accounts document that Māori society transformed from relatively autonomous village-based groups into larger territorial lineages, which later formed even larger geo-political tribal associations. These shifts have not been well-documented in the archaeological record, but social network analysis (SNA) of pXRF sourced obsidian recovered from 15 archaeological sites documents variable levels of similarity and affiliation. Three site communities and two source communities are defined based on the differential proportions of obsidian from 13 distinct sources. Distance and travel time between archaeological sites and obsidian sources were not the defining factors for obsidian source selection and community membership, rather social considerations are implicated. Some archaeological sites incorporated material from far off sources, and in some instances geographically close sites contained material from different sources and were assigned to different communities. The analytical site communities constitute relational identifications that partially correspond to categorical identities of current Māori iwi (tribal) territories and boundaries. Based on very limited temporal information, these site communities are thought to have coalesced sometime after AD 1500. By incorporating previously published and unpublished data, the SNA of obsidian artefacts defined robust network communities that reflect differential levels of Māori interaction and affiliation.

## Introduction

The Polynesian colonists who settled New Zealand some 700 years ago [1] brought with them cultural conceptions of chiefdoms based on genealogical affiliation and territoriality [2]. It has been suggested that the initial settlers lived in relatively autonomous villages [3] [4], and over several centuries these groups grew and formed geographically larger social units referred to as hapū [5]. Genealogical and historical evidence suggests hapū membership was organized through kinship and spatially referenced to particular rohe (territory). At certain times and places hapū were dynamic and non-exclusive in nature [6]. Although there was considerable variation, historic evidence suggests hapū affiliation provided access to resources, exchange opportunities, and security. By the 18th century historic evidence suggests hapū had often coalesced into larger social groups known as iwi [7] [8]. Iwi affiliation potentially offered greater security against aggressors, but competition and infighting among aligned hapū was also common. While some of these social transformations are well attested in recorded oral traditions and historical accounts, they are not well documented in the archaeological record. To investigate changes in Māori interaction and affiliation we focus on tracing the spatial and temporal distribution of artefacts made of obsidian, an important stone resource that was used for a variety of tools [9] [10].

Archaeologists have long recognized that people obtained and exchanged materials for economic reasons, with more recent substantivist approaches acknowledging the socially embedded nature of material flow [11]. Similarities and differences in artefacts are often used as indicators of the frequency or intensity of contact and relationships between groups [12] [13] [14], shared practices, or identity [15]. Exchanges reinforced “…social relationships between individuals, between an individual and his or her group, and between a group and its neighbors” [16]. The focus of many archaeological models of trade and exchange has been inter-societal as opposed to intra-societal dynamics, but with the incorporation of notions of agency and practice [17] [18] it is possible to derive interpretations of how groups within larger social units existed. While social exchange relationships are created by individuals set within cultural constructions, such relationships often had implications for the survival and persistence of groups within challenging environments [19] [20]. Understanding Māori interaction and social affiliations necessitates empirically documenting past relations and evaluating shifts in emphasis from least-cost economic considerations to socially enmeshed associations.

In Aotearoa New Zealand, obsidian is particularly well suited for studying interaction as it was widely used and limited to 27 natural sources [21]. The movement of obsidian was often the result of intentionally selecting and transporting source material, but was also a by-product of people’s mobility for other unrelated reasons [22]. Most obsidian tools were fashioned and used for short periods of time on an *ad hoc* basis, with unused debitage flakes being produced. Very few “formal” obsidian tools (e.g., adzes, drill-points) are ever archaeologically recovered; the overwhelming majority of obsidian artefacts are unstandardized or “informal” flake tools [9] [10]. Replication studies suggest that these tools were used in a variety of ways as an all-purpose cutting and scraping tool, particularly for the working of flax, butchering, and woodworking [9] [23] [24]. The informal nature of obsidian artefacts distinguishes them from other more formal tools, such as basalt adzes [10]. This informal nature is analytically advantageous because social factors and matters of expediency might have often played more significant roles than the quality of obsidian in choosing source material.

Obsidian sourcing studies have noted a general trend for Mayor Island obsidian to dominate site assemblages, particularly in early sites predating the 17^th^ century [21] [25] [26] [27]. Mayor Island is located some 35 km off the Coromandel Peninsula, and was a major source of obsidian distributed throughout the country. The differential proportions of Mayor Island and other obsidian sources has been interpreted as reflecting interaction, population mobility, and social and genealogical affiliations [27] [28] [29]. While social factors were important, several studies [28] [30] suggest that the location of a site, whether it was inland or on the coast, and the distance of a site from a source, influenced the proportions of source material. At inland sites, McCoy et al. [31] proposed that ‘procurement areas’ were quite small, with a range of some 30-50km, whereas coastal sites contained greater proportions of distant source material due to the ease of access to those sources via canoe travel.

Documenting patterning in the spatial distribution of obsidian has been enhanced by recent advances in social network analysis (SNA) that utilize models and quantitative measures to study interaction and social affiliation [14] [32] [33] [34] [35]. These models represent entities, such as artefacts, archaeological sites, or natural obsidian sources, as a series of ‘nodes’. These nodes can be assigned attributes, such as the proportion of obsidian from different natural sources found at a particular archaeological site, or the estimated age of an artefact. Relationships between nodes are represented as edges or links and can also be assigned properties, such as the strength of similarities between pairs of nodes. Multipartite networks allow for networks that consist of multiple different node types with links only allowed between nodes of different types. For example, node types could be artefacts, archaeological sites, and obsidian sources, with links indicating when an artefact from a specific source was found at a particular site. SNA focuses on both the nodes and the relationships between nodes, with the understanding that the properties of the system are an aggregate of both. Archaeologists in other parts of the world have analysed obsidian distributions with SNA to study a range of processes including exchange [36] [37], procurement methods [38], interaction spheres and social affiliation [32] [39] [40], mitigating risk in unpredictable environments [20] [41], and societal collapse [42].

In the following we use SNA to investigate Māori interaction and affiliation by analysing obsidian artefacts made from various source materials found at archaeological sites. Our analysis incorporates data on obsidian artefacts collated from four previous pXRF obsidian sourcing studies in the Auckland area and Northland [21] [29] [30] [31]. We also incorporate data summarized in a regional database [43][44] that includes data from Kneebone [26] and Cruickshank [25] and new data from Great Mercury Island, Coromandel Peninsula. We use cosine metrics to define similarities between archaeological sites based on the proportions of obsidian artefact source material found at the sites. These similarities allow us to define “communities”, sets of nodes whose members are more similar to each other than nodes (i.e. archaeological sites) of other sets. The spatial and temporal patterning in these communities is assessed in terms of least-cost travel between archaeological sites and sources to evaluate the extent to which people utilized obsidian in proportion to the distance and cost of travel to sources, and considerations of social affiliation and levels of interaction.

## Materials and methods

**Fig 1:**
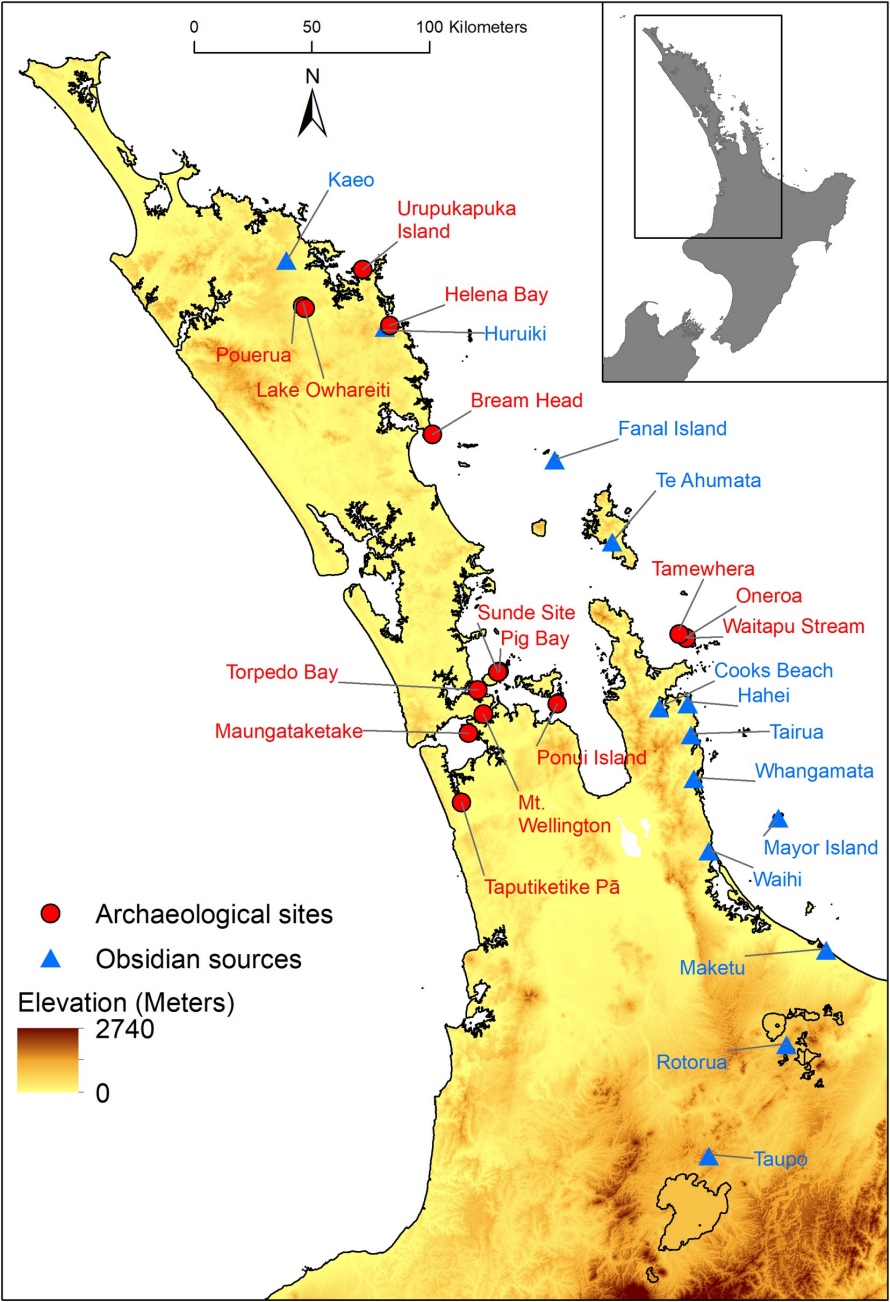
The 15 archaeological sites and 13 obsidian sources. Contains data sourced from the LINZ Data Service licensed for reuse under CC BY 4.0.

The combined data set comprises 2404 obsidian artefacts from 13 distinct sources recovered from 15 archaeological locations (Table 1; Fig 1; S1 Table). In nearly all cases these locations correspond to a single site number in the New Zealand Archaeological Association’s geodatabase of archaeological sites (www.archsite.org.nz), and we use the term site to refer to these locations. The temporal associations of the sites vary, and for 13 of these, we determined whether the assemblages from the sites likely pre or post-date AD 1500 based on the reported radiocarbon dates (see Table 1, and [21] [25] [29] [30] [43]). The largest assemblage from a site, in terms of the number of artefacts, is Ponui Island with 565 artefacts, while the sites with the smallest assemblages are Taputiketike and the Sundae site, with 20 and 22 artefacts, respectively. As noted above, Mayor Island was often a major source of obsidian found in archaeological sites, and is by far the dominant source of material in the data set, with 915 artefacts, almost twice the number of the next most prolific source; Kaeo with 501 artefacts. The rarest sources, Rotorua, Taupō, and Tairua contribute only two, two, and five artefacts, respectively.

**Table 1:** The frequency of artefacts found at each archaeological site.

Site Name	NZAA number	Reference	Temporal association	Cooks Beach	Fanal Island	Hahei	Huruiki	Kaeo	Maketu	Mayor Island	Rotorua	Tairua	Taupo	Te Ahumata	Waihi	Whangamata	TOTAL
**Bream Head**	Q07/78; Q07/774; Q07/747	**McCoy and Carpenter (2014)**	**post AD 1500**	0	12	0	1	0	0	15	0	0	0	5	0	0	33
**Helena Bay**	Q05/567	**McCoy and Carpenter (2014)**	**post AD 1500**	0	0	0	329	0	0	0	0	0	0	0	0	0	329
**Lake Owhareiti**	P05/857	**McCoy et al (2014)**	**pre AD 1500**	0	3	0	11	40	0	0	0	0	0	0	0	0	54
**Maungataketake**	R11/31	**Kneebone (2016)**	**Unknown**	8	0	10		0	0	155	0	0	0	134	0	4	311
**Mt Wellington**	R11/12	**McCoy and Carpenter (2014)**	**post AD 1500**	4	5	3	1	0	0	15	1	0	0	96	0	0	125
**Oneroa**	T10/944	**McCoy et al. (2019)**	**pre AD 1500**	5	0	2	1	0	0	31	1	0	0	0	0	0	40
**Pig Bay**	R10/22	**Cruickshank (2011)**	**pre AD 1500**	2	0	5		0	0	55	0	0	0	0	0	4	66
**Ponui Island**	S11/20	**Sheppard et al. (2011)**	**pre AD 1500**	4	2	92	1	0	31	382	0	5	2	35	10	1	565
**Pouerua Pa**	P05/195	**McCoy et al (2014)**	**pre AD 1500**		2		28	181		4							215
**Pouerua Pa**	P05/195	**McCoy et al (2014)**	**post AD 1500**				13	268		6				1			288
**Sunde Site**	R10/25	**Cruickshank (2011)**	**pre AD 1500**	0	0	10		0	0	10	0	0	0	0	0	2	22
**Tamewhera**	T10/214	**McCoy et al. 2019)**	**post AD 1500**	20	0	11	1	0	0	153	0	0	0	1	0	0	186
**Taputiketike Pa**	R12/348	**Cruickshank (2011)**	**post AD 1500**	0	0	0		0	0	4	0	0	0	16	0	0	20
**Torpedo Bay**	R11/1945	**Cruickshank (2011)**	**pre AD 1500**			2				21						5	28
**Torpedo Bay**	R11/1945	**Cruickshank (2011)**	**post AD 1500**					1		1				1			3
**Torpedo Bay**	R11/1945	**Cruickshank (2011)**	**Unknown**							1				1		9	11
**Urupukapuka Island**	Q05/1101; Q05/1070	**McCoy et al. (2010)**	**Unknown**	4	0	0	7	11	0	19	0	0	0	0	0	0	41
**Waitapu Stream**	T10/360	**McCoy et al. (2019)**	**pre AD 1500**	14	0	9	1	0	0	43	0	0	0	0	0	0	67
**TOTAL**		** **	** **	61	24	144	394	501	31	915	2	5	2	290	10	25	2404

Focusing on the frequency of artefacts at a site might not produce an accurate reflection of the total amount of obsidian procured from various sources. Using information on mass, as opposed to frequency, may be desirable as the mass of artefacts mitigates the analytical impact of large numbers of low mass flakes that are potentially produced and discarded at a site when working obsidian. However, the obsidian artefacts analysed in our study are generally larger than 20mm, a common cut-off in lithic artefact studies [45] [46]. Using this cut-off limits the potential impact of incorporating large numbers of low mass flakes into a frequency based analysis. Experimental studies have also indicated that the overall volume of material unanalysed as a result of only analysing artefacts larger than 20 mm is negligible [47] [48]. Further, using a >20mm cut-off reduces biases caused by post-depositional processes as artefacts larger than 20mm have a lower potential for movement [49] [50]. Of the 2404 artefacts in our analysis, 1354 have estimates for their individual weight. We use similarity indices to evaluate the difference between using frequency as opposed to mass for defining social networks.

### Bipartite networks

**Fig 2:**
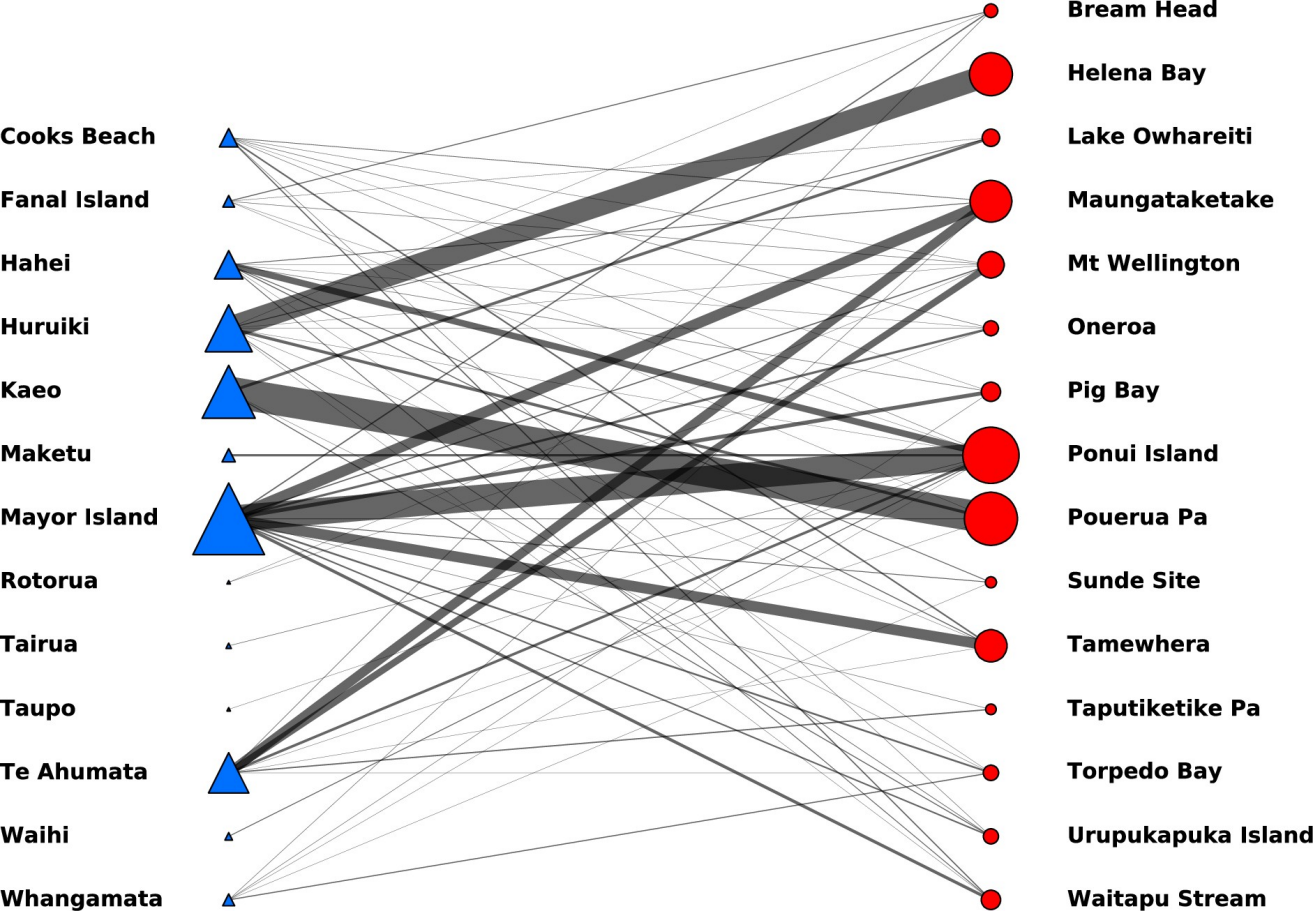
A bipartite network of obsidian sources and archaeological sites, with links (edges) indicating the frequency of artefacts associated with each source-site pair.

The frequency matrix of sites and sources (see Table 1) defines a bipartite, or two-mode, network. A bipartite network is a network with two distinct sets of nodes–in this case sources and study sites. Edges, or links, in the network can connect nodes of different types, but not nodes of the same type. In this case, edges represent artefacts originating from a particular source and found at a specific study site. Weights can be associated with each edge to indicate, for example, the number of artefacts, or the total mass of artefacts associated with each source-site pair. Fig 2 shows such a network. In this case, the size of the nodes in the network are proportional to the number of artefacts recorded at, or sourced from, each study site, or source location. Similarly, the thickness of the edges in the network indicate the number of distinct artefacts associated with each source-site pair.

Information on the weight of individual artefacts was available for a subset of the data (1354 of the 2404 total artefacts; see S1 Table). For these cases, we constructed a network similar to that represented in Fig 2, but with node sizes and edge weights proportional to the mass of artefacts (S1 Fig). Only 56% of the total artefacts in the data sets have mass information recorded, reducing the size and potentially the significance of the mass data available for study. We estimate the consequences of using or not using the mass information by calculating a mass-frequency similarity measure.

### Similarity

We make use of similarity calculations in two different contexts: to quantify the similarity between two *different* types of measurements (e.g., mass and frequency) of the *same assemblage* of artefacts; and to quantify the similarity between two *distinct assemblages* for a *single type of measurement* (e.g., source frequency counts for artefacts from two different sites). The approach to calculating both of these similarity measures is mathematically the same but, for clarity, we distinguish between the measurements, when necessary, by referring to them as self-similarity and cross-similarity, respectively.

The *cosine self-similarity* between, for example, the frequency and mass data at a site *A* is given by ${Sim}_{A}=\cos\theta =\frac{u_{A}∙v_{A}}{\left\|u_{A}\|\|v_{A}\right\|}$ where *u*_*A*_ is a vector of measurements of type *u* (e.g., the number of artefacts from each source), found at site *A*, *v*_*A*_ is a vector of measurements of type *v* (e.g., the total mass of artefacts from each source), and *u*∙*v* is the vector dot product. In this context, the cosine similarity can be interpreted as the cosine of the angle *θ* between two vectors, the direction of which is determined by the relative abundances of the mass or frequency of artefacts from each source. A similarity value of one indicates identical relative abundances of artefacts as measured by mass and frequency, while a value of zero would indicate complete dissimilarity (i.e., orthogonal abundance vectors).

The *cosine cross-similarity*, for a measurement of type *u* at sites *A* and *B* is given by ${Sim}_{A,B}=\cos\theta =\frac{u_{A}∙u_{B}}{\left\|u_{A}\|\|u_{B}\right\|}$.

### Bipartite network projections

Given a bipartite, or two-mode network, one is often interested in inferring the relationships between one of the node types in the network. Projecting the bipartite network results in a one-mode network, with edges indicating relationships between nodes according to the projection method used. In the simplest case a one-mode projection might connect nodes of the same type whenever they share a common neighbour of the opposite node type in the bipartite network. We project the bipartite source-site network onto both the site and source nodes, using the cross-similarity between the assemblage frequency vectors, for pairs of sites (respectively sources), to define the edge weights of the projected site (respectively, source) similarity networks. This allows us to define sets of archaeological site communities, and sets of source communities.

### Least-cost paths between archaeological sites and obsidian sources

We used least-cost path analysis (see [51] [52] [53] for recent studies) between archaeological sites and obsidian sources to assess whether material recovered at archaeological sites was in proportion to distances or “costs” between sites and sources. Our null hypothesis is that people directly accessed the closest, or easiest, obsidian sources in terms of travel time to procure lithic material. We calculated least-cost paths between sites and obsidian sources using the “NZ 8m Digital Elevation Model (2012)” dataset downloaded from Land Information New Zealand Toitū te Whenua (https://www.linz.govt.nz/data/linz-data) that we reduced to a 100m resolution to facilitate data processing. We used Tobler’s [54] ‘hiking function’ to calculate the time it would take to complete trips from all sites to all sources. We implemented the least-cost path analysis within ESRI ArcMap 10.6 using a Tobler vertical factor table downloaded from http://mapaspects.org/node/3744/.

We evaluated whether the least-cost paths between archaeological sites and obsidian sources changed if travel over water in relation to travel over land was considered relatively easy or relatively difficult. It could be assumed that travel over water was more “costly” due to inherent risks of sailing or paddling over the ocean, or alternatively, it could be assumed that canoe travel was less “costly” because it would be easier to transport larger quantities of obsidian in a canoe then by carrying it over land. We used the Path Distance and Cost Path tools of ESRI ArcMap 10.6 to implement a series of results where the cost of travel over the ocean was incrementally increased by 0.1 in relation to the cost of travel over flat land. We found that when travel over the ocean was the same as travel over flat land the routes between archaeological sites and obsidian sources maximized the time and distance travelled over the ocean (see S2 Fig for an example). If the cost of travel over the ocean was increased to 1.2 times the cost of travel over flat land, the routes increased in terms of travel over land in relation to the ocean. When ocean travel costs were increased to twice that of travel costs over flat land the routes shifted to maximizing travel over the land (see S3 Fig for an example). While increasing the cost of travel over the ocean in relation to the cost of travel over flat land resulted in substantial changes in travel time and the routes that were defined, it is notable that these increases only produced minor changes in the rank order of least-cost paths between archaeological sites and obsidian sources (S2 Table).

## Results

### Mass versus frequency data

We quantified the effect of using frequency versus mass edge weightings for the links in the bipartite network by calculating the cosine self-similarity between the mass and frequency data for each study site. For those assemblages for which mass data was available, there was an extremely strong similarity between the relative abundances of material, whether measured by mass or frequency, indeed the lowest mass-frequency self-similarity value was 97.89% (S3 Table). Because of the high correlation between the two data sets, and the larger size of the frequency data (n = 2543 vs. n = 1354), our subsequent analyses focus on the frequency data.

### Site similarity communities

To establish communities of like sites and communities of like sources, we used the cosine cross-similarity to project the bipartite site-source network onto each of its node types, creating the site and source one-mode networks. Within these one-mode projected networks, communities of nodes, be they sites or sources, high similarities were identified using the Louvain community detection method [55]. This community detection algorithm aims to maximize modularity, a measure of edge density within communities in relation to the number of edges between communities. By calculating the node sets that maximise this property of the network, we identified communities with the highest amount of similarity within the separate groupings.

**Fig 3:**
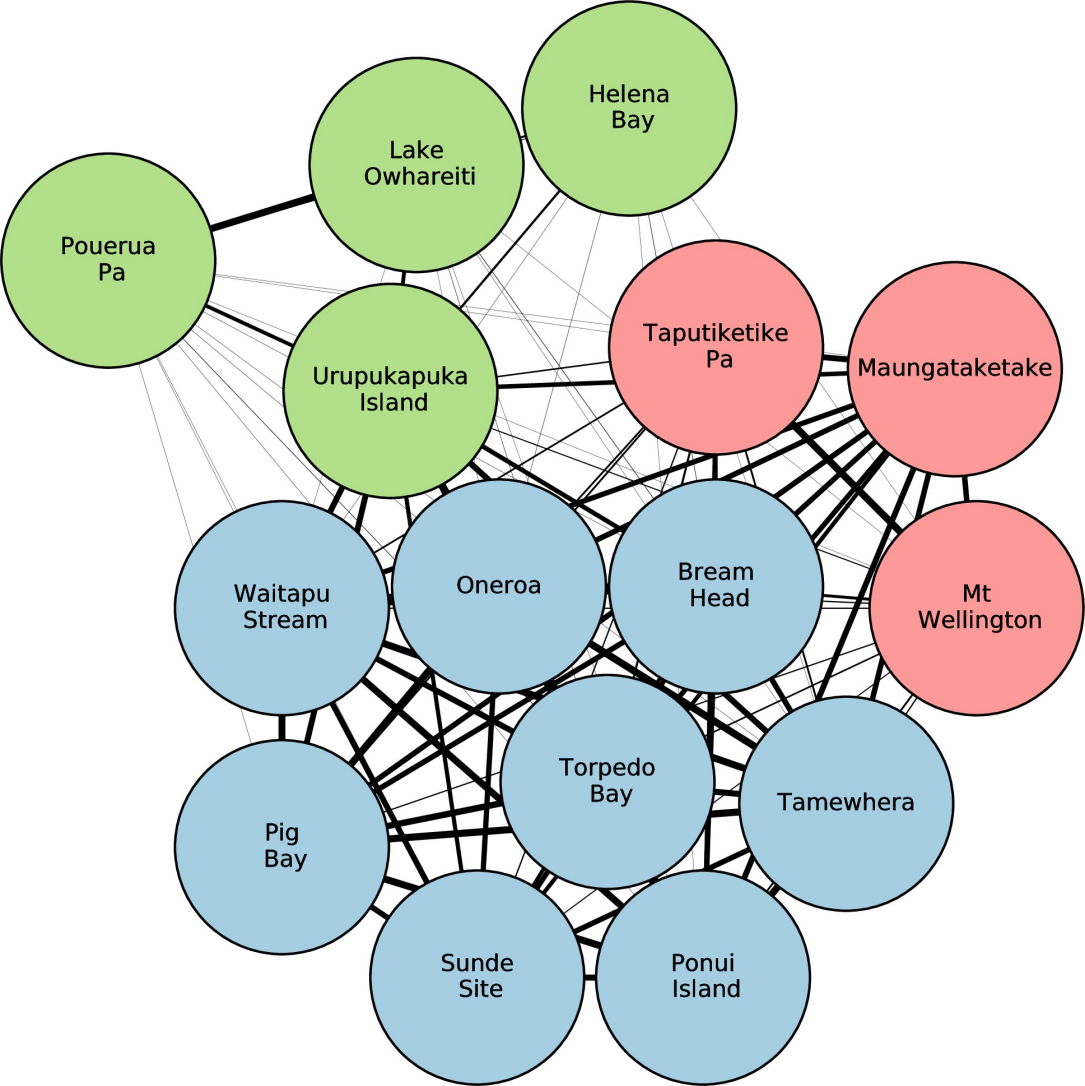
Archaeological site communities with similar proportions of obsidian from specific sources. The relative thickness of the links indicates the strength of similarity. Site Community A coloured light green; Site Community B coloured light blue; Site Community C coloured pink.

**Fig 4:**
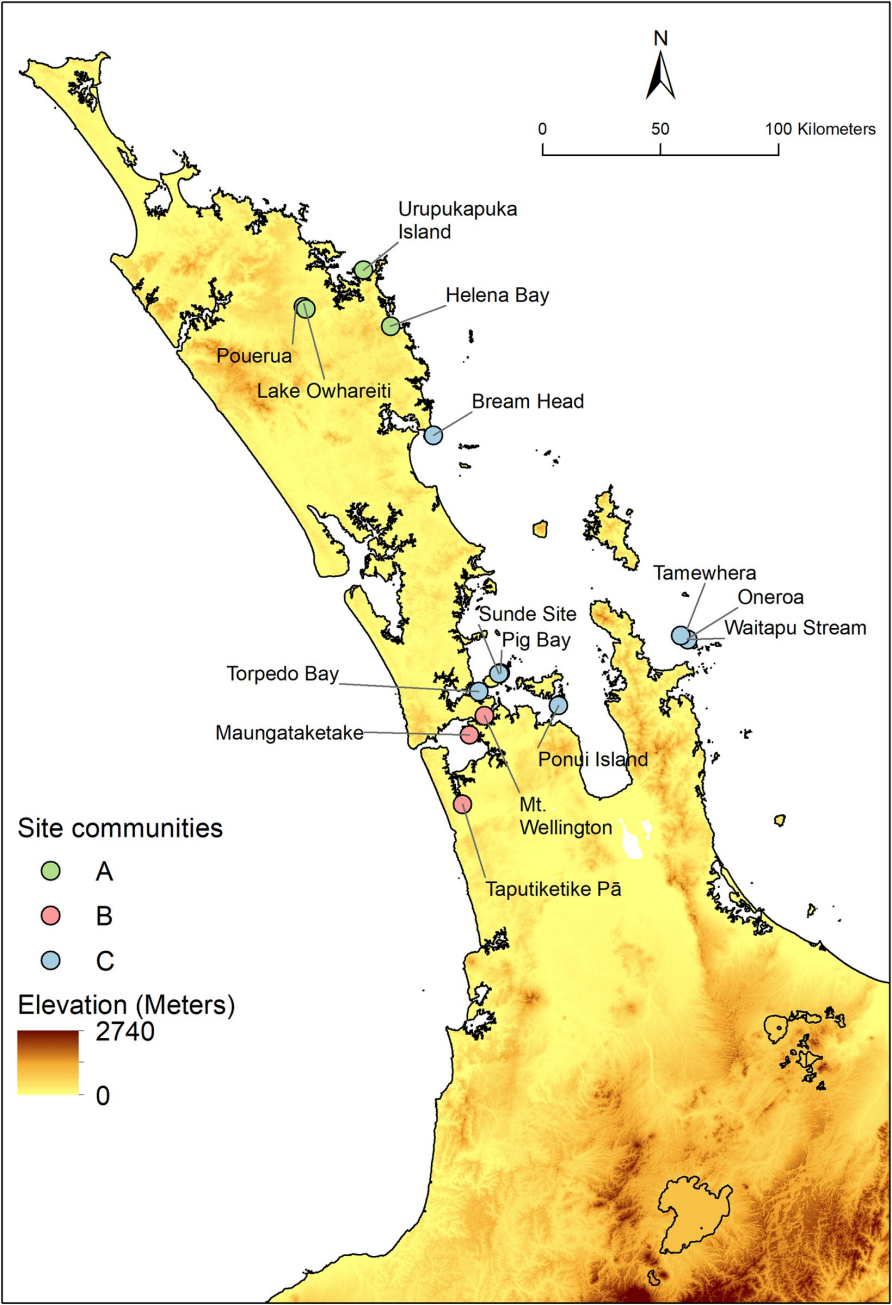
The spatial distribution of archaeological site communities. Contains data sourced from the LINZ Data Service licensed for reuse under CC BY 4.0.

The three site communities, each containing similar proportions of obsidian from specific sources, are shown in Fig 3, and these have significant spatial patterning (Fig 4). There is a distinct Northland cluster (hereafter labelled and referred to as the Full Network Site Community A, or just Site Community A), a South-West Auckland cluster (labelled Site Community B), and a North-East Auckland/Hauraki Gulf/Coromandel Peninsula cluster that also includes a spatial outlier Northland site of Bream Head (labelled Site Community C).

**Fig 5:**
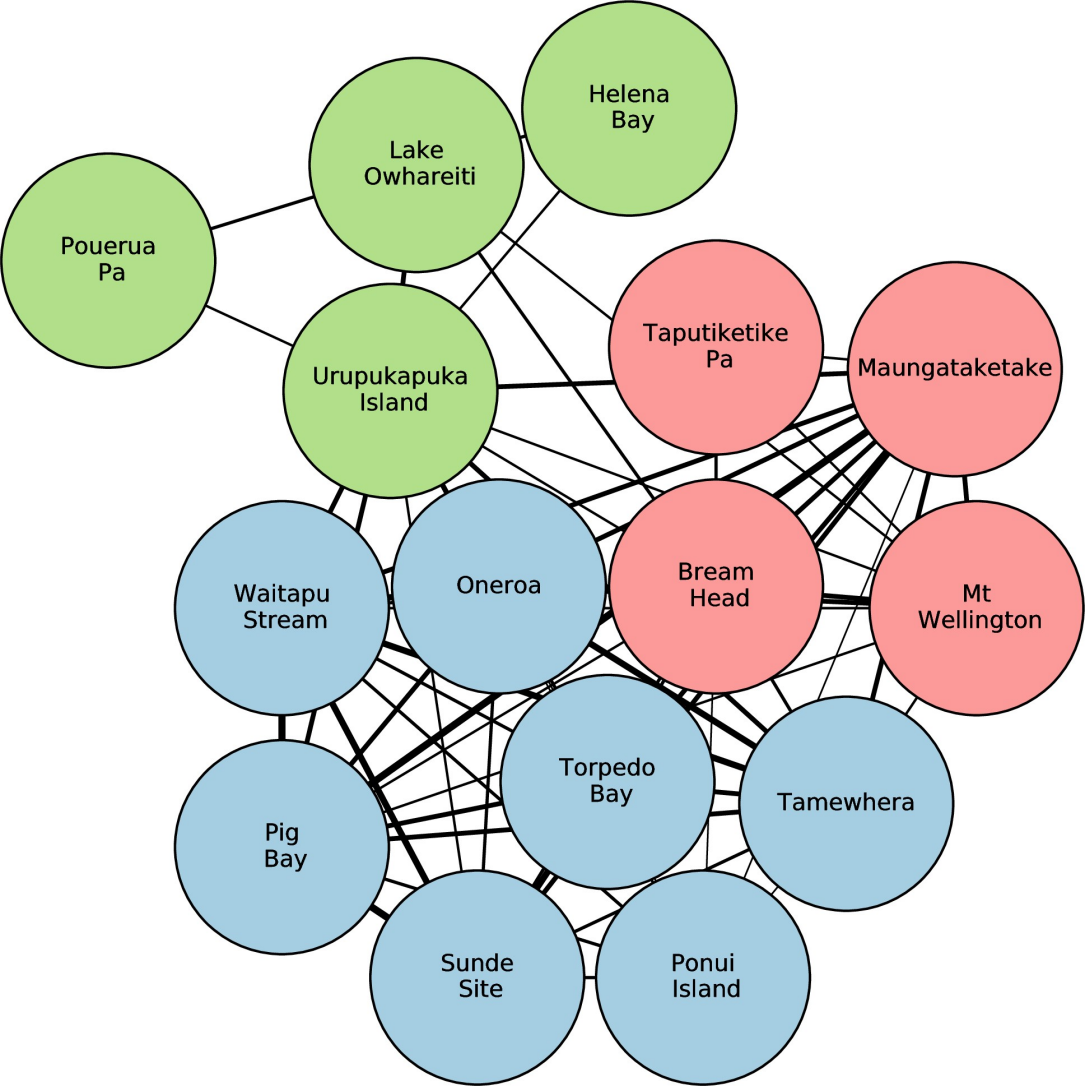
Archaeological site communities taking into account the revealed comparative abundance (RCA) of obsidian for each site-source pair. The relative thickness of the links and the spatial relationship of the nodes reflects similarity. Site Community A coloured light green; Site Community B coloured light blue; Site Community C coloured pink.

Since much of the obsidian represented in the dataset was sourced from Mayor Island, we wished to assess whether this source material in the sample was affecting the community structure observed in Fig 3. To do so we calculated the revealed comparative abundance (RCA) of obsidian for each site-source pair. The RCA method is based on the method of revealed comparative advantage, originally used in economics [56] [57] [58]. It considers the amount of obsidian for a site-source pair, accounting for both the amount of obsidian from that source, relative to that from all other sources, and the amount of obsidian found at that site, relative to the amounts found at other sites. Such an analysis on the original dataset therefore adjusts the weights of the edges in the bipartite network to take account of the frequency of the material in the entire network. The result of the projected site network for the RCA analysis (Fig 5) is very similar to the original projected site network (see Fig 3). Bream Head is the only archaeological site that changes site communities in the RCA analysis, shifting from Site Community C to Site Community B. The weightings of several edges in the RCA network have changed and some edges from the original network are no longer present in the RCA network, however the overall structure of the network is largely unchanged—as indicated by the fact that the site communities identified in the network by the Louvain algorithm remain very similar to those identified in the original site similarity network. This leads us to conclude that the high frequency of Mayor Island obsidian is not obscuring the community detection patterning.

### Bipartite network of site and source communities

**Fig 6:**
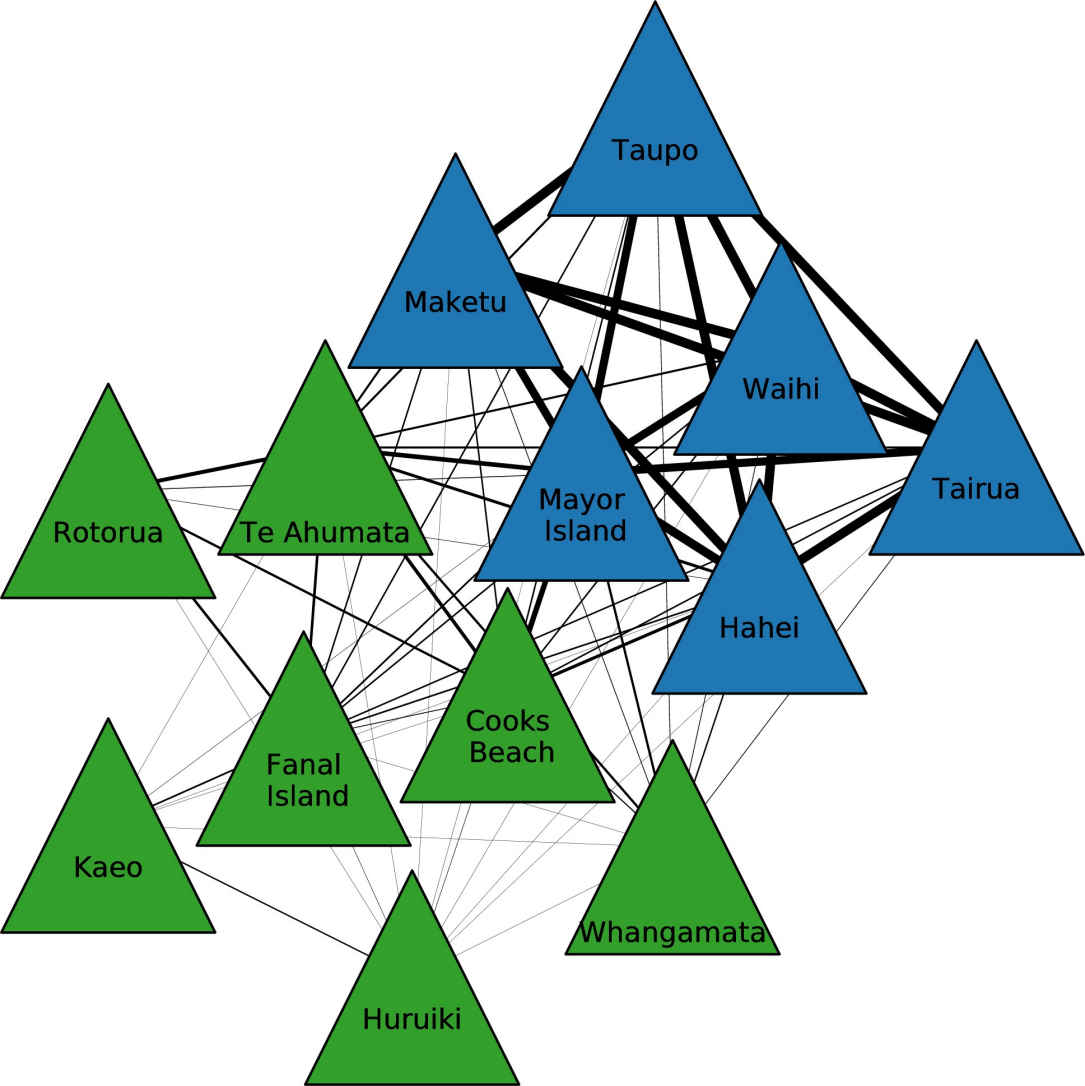
Obsidian source communities. The relative thickness of the links and the spatial relationship of the nodes reflects similarity. Source Community 1 coloured dark green; Source Community 2 coloured dark blue.

**Fig 7:**
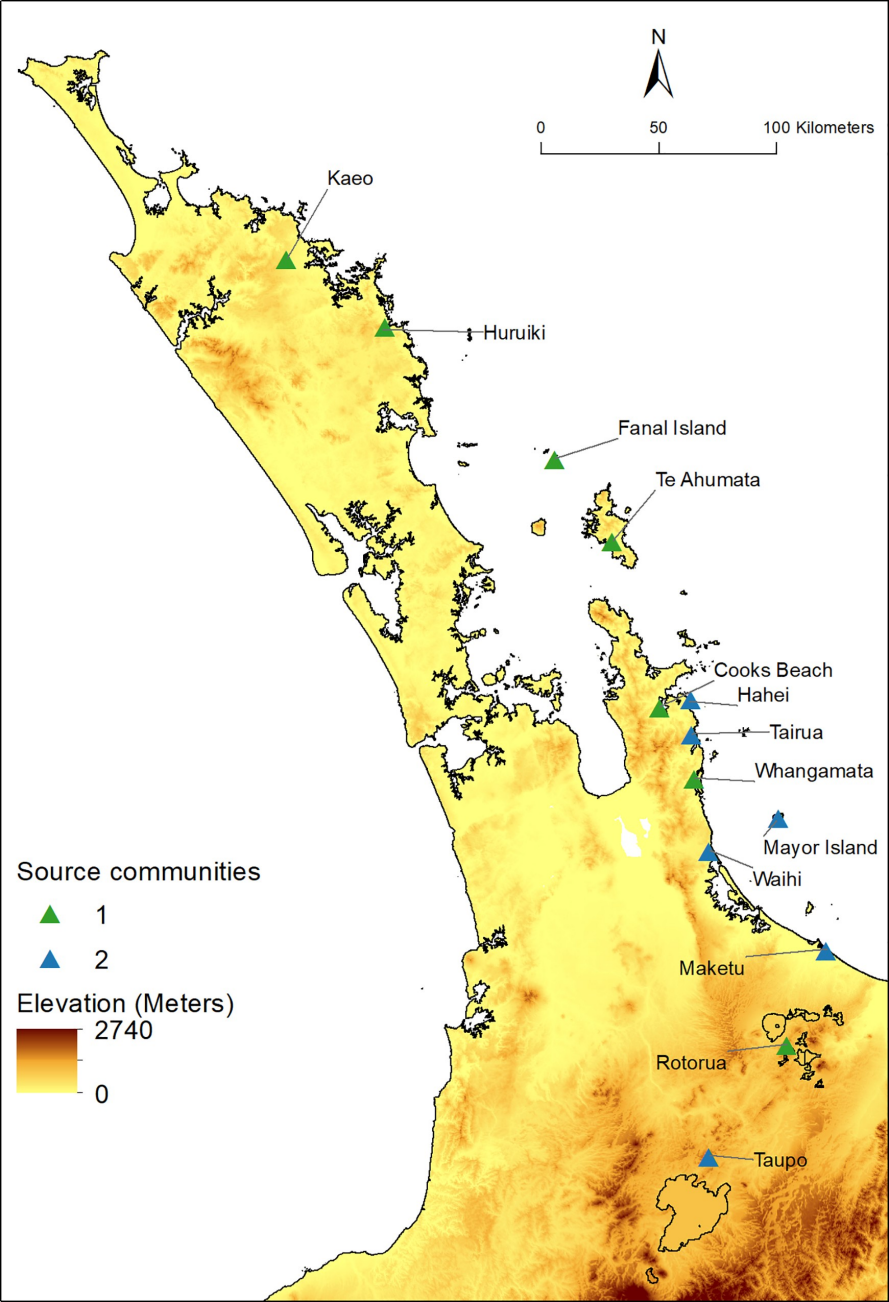
The spatial distribution of obsidian source communities. Contains data sourced from the LINZ Data Service licensed for reuse under CC BY 4.0.

We ran the same analysis, with cross-similarity projection and community detection methods, for obsidian sources to identify which sources were most similar to other sources in terms of being a supply of material for particular sites. This resulted in the identification of two source communities (Fig 6) which had distinct spatial patterning (Fig 7). The first, referred to and labelled as the Full Network Source Community 1, or just Source Community 1, includes Northland sources of Kaeo and Huruiki, the Fanal Island source, the Te Ahumata source on Great Barrier Island, the Whangamata and Cooks Beach Coromandel Peninsula sources and the further afield Rotorua source material. The second, labelled Source Community 2, includes a more spatially clustered community of sources based in the Coromandel Peninsula and the Bay of Plenty (Hahei, Maketu, Tairua, Waihi), Mayor Island, and Taupō.

**Fig 8:**
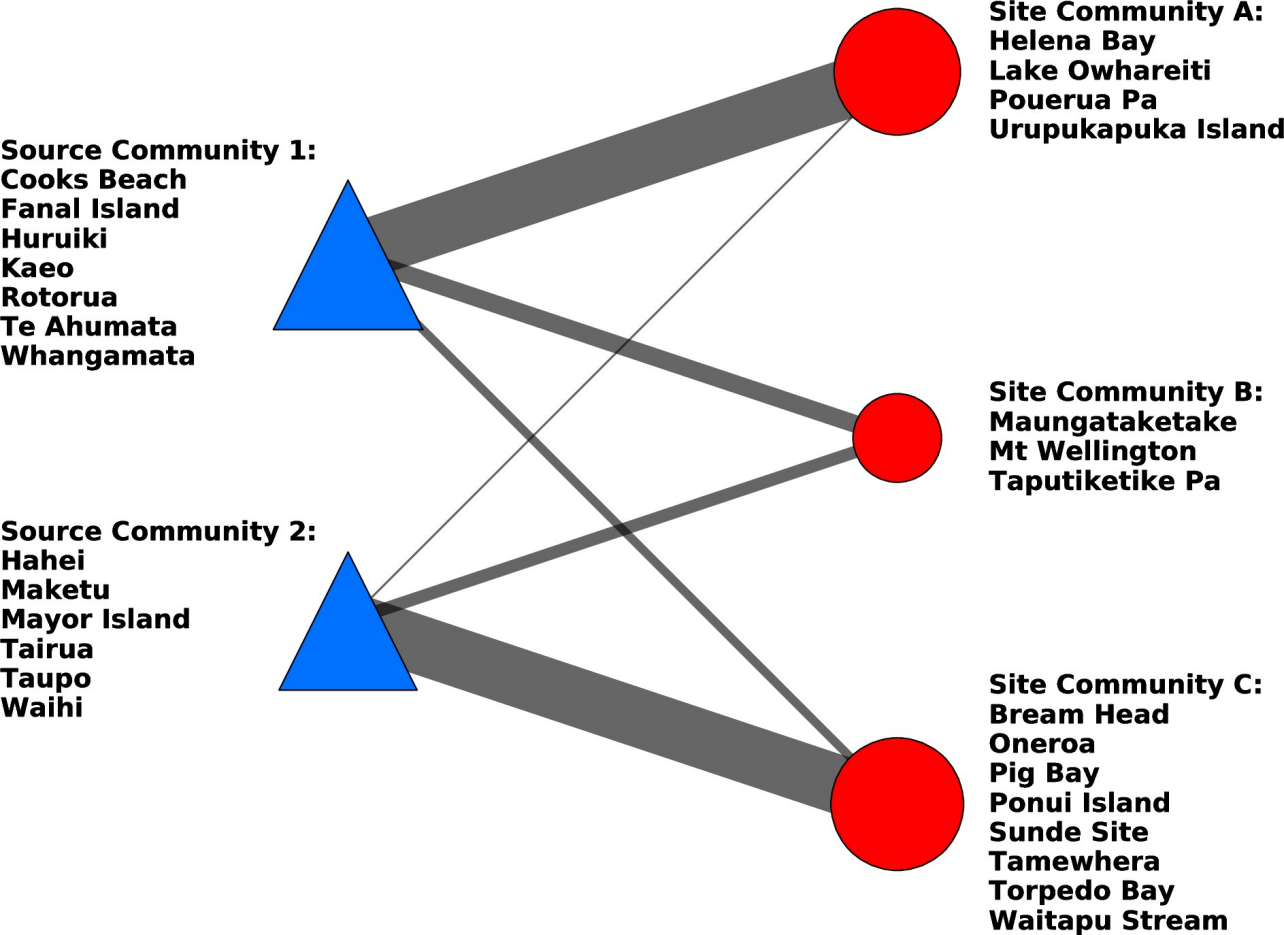
A bipartite network of obsidian source communities and archaeological site communities, with links indicating the frequency of artefacts associated with each source-site pair.

The bipartite network for the combined site-source communities is shown in Fig 8. Two of the site similarity communities identified tend to procure their obsidian from single, reasonably well defined source similarity communities. Site Community A procures most of its obsidian from Source Community 1, and Site community C procures most of its obsidian from Source Community 2. In contrast, Site Community B procures material from Source Communities 1 and 2 in roughly equal proportions.

### Robustness of similarity communities

To analyse the statistical significance, or robustness, of the community structures identified in the site and source community detection analyses, we tested how the full community bipartite graph (see Fig 8) changed when the data underlying it was perturbed. To simulate the effect of incorrectly categorised artefacts or other data collection errors, we added further artefacts to our original bipartite network, randomly assigning them site and source connections. We recalculated the community structure to evaluate whether or not it changed with the addition of the new artefacts. At each level of noise (number of added artefacts) we carried out 1,000 simulations and calculated an average difference value for the number of nodes which were assigned to a different community, along with the corresponding standard deviations. Typically, 700 additional, randomly assigned artefacts needed to be introduced to the network before the network structure is perturbed sufficiently for a site node to be assigned to a different community (S4 Fig). With the introduction of these fictitious artefacts, Bream Head was the most likely site to change communities (S5 Fig). The less certain nature of Bream Head coincides with the results from comparing the RCA projected site network with the original projected site network, where Bream Head changed communities. The introduction of 700 randomly assigned artefacts in the network amounts to adding around an additional 29% of random data, suggesting that the observed structure of the bipartite network, and the associated communities are indeed robust and significant.

### Spatial distribution of site and source communities

**Fig 9:**
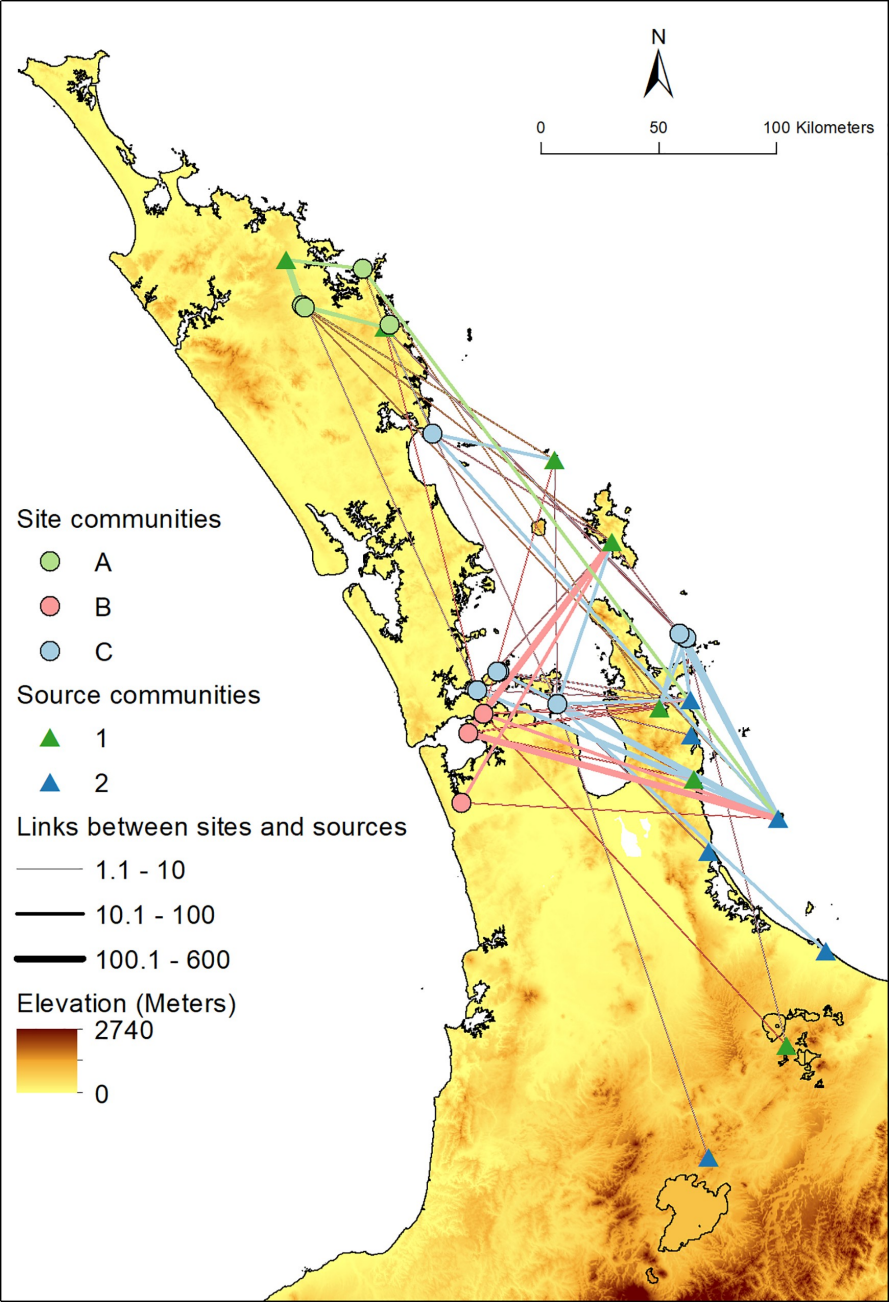
The spatial distribution of obsidian source communities and archaeological site communities. The links between sites and sources are colour coded according to the sites. The weighting of the links reflects the frequency of artefacts found at that site made out of the source material. Contains data sourced from the LINZ Data Service licensed for reuse under CC BY 4.0.

The site and source communities identified in Fig 8 are to some extent spatially clustered, though there are a number of notable long range links between site and source nodes that are revealed when the bipartite community network is embedded geographically (Fig 9). The sites in the Northland Site Community A (light green circles) are characterized by receiving obsidian from the relatively nearby sources of Source Community 1 (dark green coloured triangles), with lesser amounts of material from further afield sources on Great Barrier Island, Coromandel Peninsula, and Mayor Island. The Site Community C sites (light blue circles) are characterized by generally containing material from the Source Community 2 sources in the Coromandel Peninsula, Bay of Plenty and a small amount of material from Taupō (dark blue triangles). In contrast, the sites that comprise Site Community B (pink circles) are characterized by material from far off Source Community 1 sources such as Te Ahumata (Great Barrier Island), Whangamata, and Rotorua, with additional material from Source Community 2 sources, predominantly Mayor Island.

One possible explanation for the observed site communities is that they arose from people procuring obsidian from the sources that were most conveniently located to the sites. We tested this by deriving the expected distribution of obsidian that would be observed under an assumption of least-cost travel from sites to sources. On average, the archaeological sites obtained obsidian sourced from three locations. If obsidian patterns were solely based on geographic location, we would expect the three sources to be those that took the least amount of time to travel to and from. The rank order of least-cost travel from all sites to all sources for the two different scenarios (the cost of travel over the ocean being equivalent to travel over a flat land; and the cost of travel over the ocean being equivalent to twice that of travel over flat land) is given in S2 Table. At the Northland sites of Helena Bay and Lake Owhareiti the rank order of obsidian frequency from sources and the least-cost travel to sources are the same. At Pouerua Pa, the rank order of material is very similar to the rank order of the least-cost travel to sources, with the exception of the inclusion of material from Mayor Island and far off Taupō. At Bream Head and Maungataketake, the rank order of sources and least cost travel to sources is somewhat similar, with Mayor Island and one other source being out of sequence. At the other 10 archaeological sites, the rank order of sources and least cost travel to sources is far more disparate. In these cases it implies that social considerations, as opposed to simple geographic distance and travel time, were more important, specifically for generating the split between Community 2 sites and Community 3 sites.

### Temporal patterning in site and source communities

**Fig 10:**
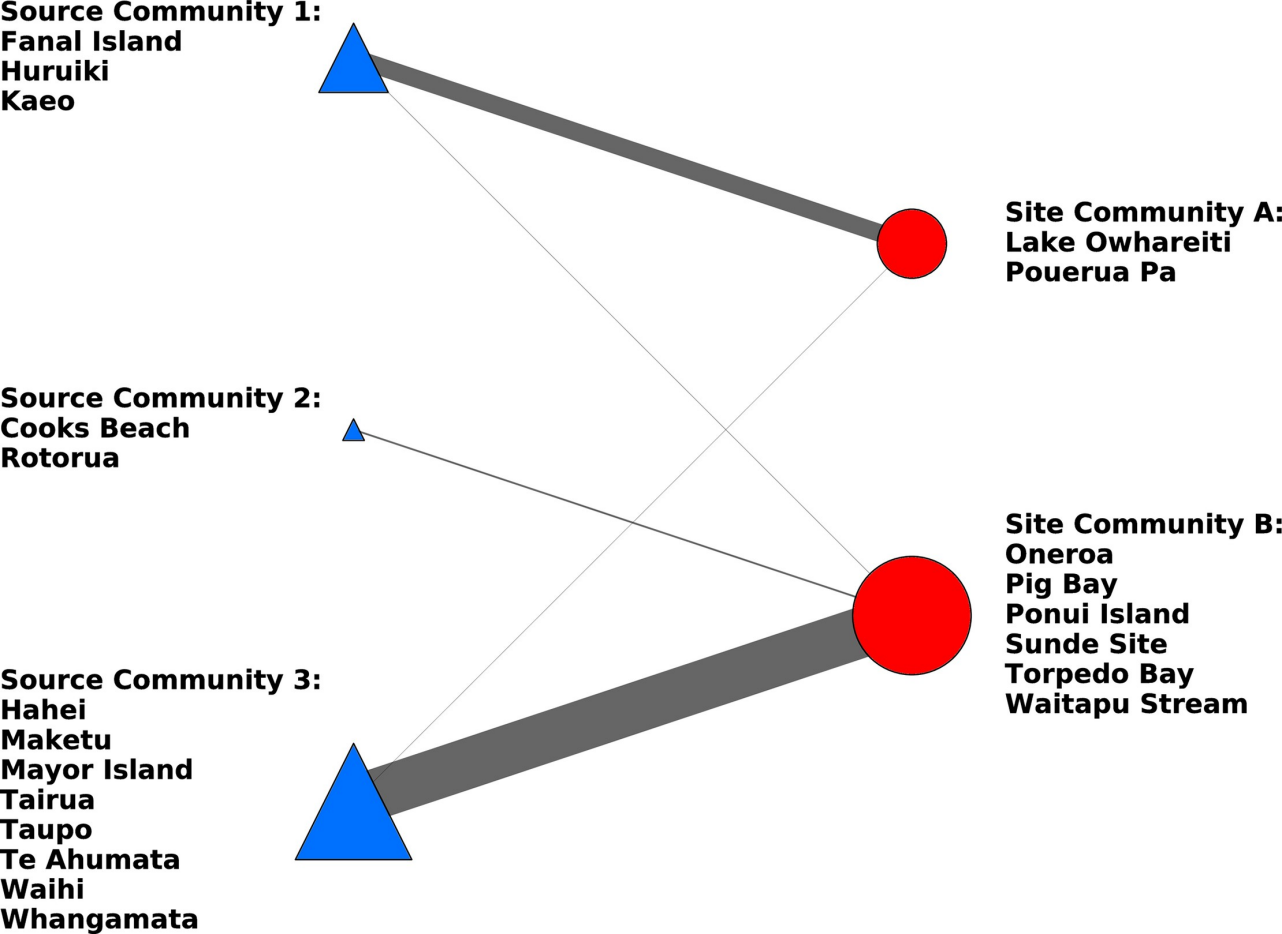
Early sites bipartite network of obsidian source communities and archaeological site communities. Links indicate the frequency of artefacts associated with each source-site pair.

**Fig 11:**
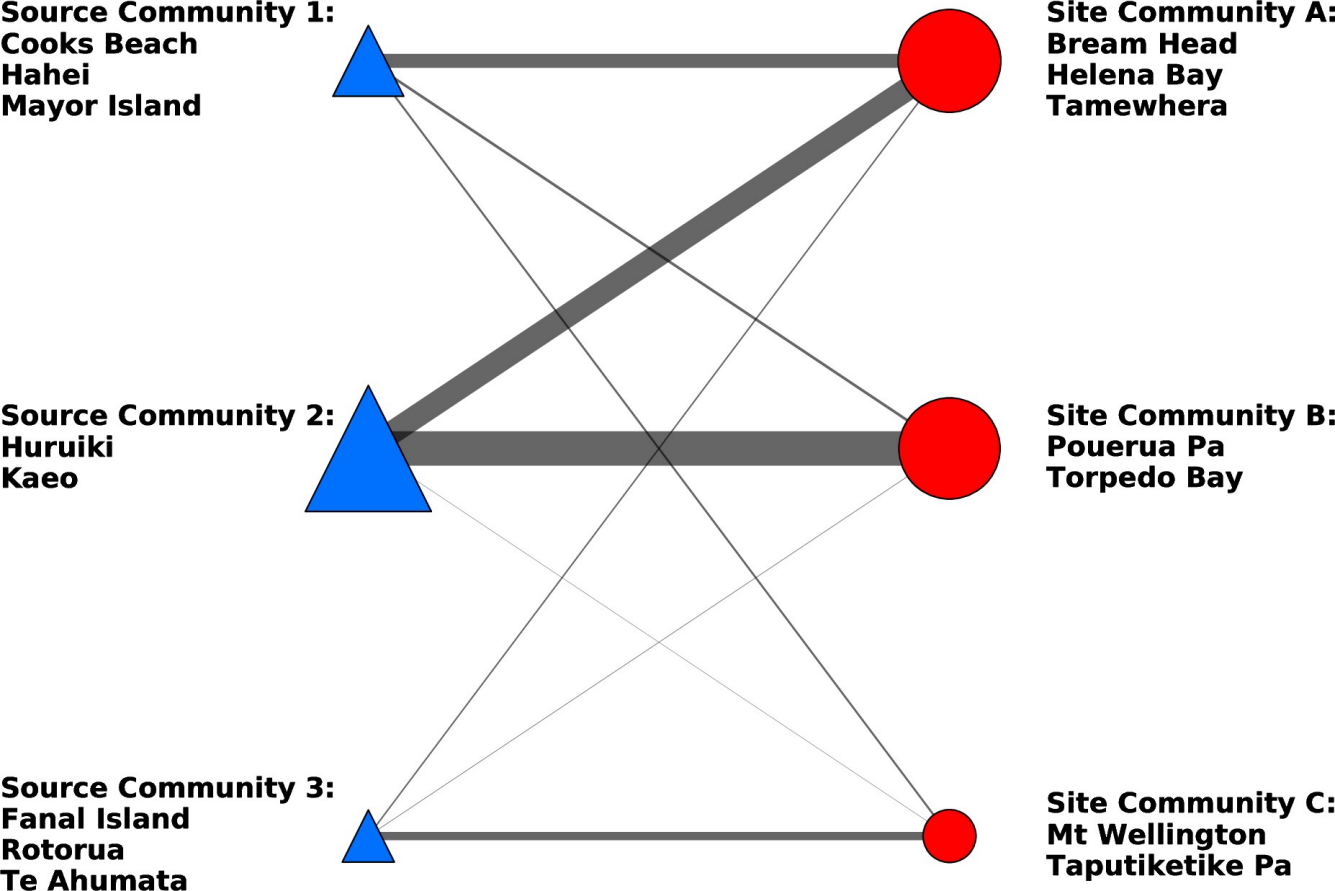
Late sites bipartite network of obsidian source communities and archaeological site communities. Links indicate the frequency of artefacts associated with each source-site pair.

The spatio-temporal patterning of obsidian use was evaluated by splitting the dataset into two smaller temporal subsets based on the associated radiocarbon dates of the archaeological sites. While the reported calibrated date ranges of the sites (see [21] [25] [29] [30] [43]) are not completely discrete, it is possible to categorize sites into those that probably predate AD1500 (Early) and those that probably postdate AD 1500 (Late). To investigate temporal patterning we created community bipartite graphs for sites in those two temporal categories (Fig 10 and Fig 11). This categorization results in much smaller sample sizes for the data sets (early sites = 8; late sites = 7) than the full network (sites = 15). Although it is not possible to draw firm conclusions from such limited data, it is notable that Full Network Site Community C identified in the original analysis is split into two site communities when just the early sites are considered. In the early site configuration, Pouerua Pa and Lake Owhareiti create one community and the other sites that were originally part of the Full Network Site Community C form a separate community (compare Figs 8 and 10). The early site analysis results in three source communities being defined, instead of the original Full Network two source communities. In this case, the original Full Network Source Community 1 is broken into two communities. In sum, reducing the sample size of sites from 15 to just include the 8 early sites creates divisions in both the full network site and source communities originally identified.

When only the late period sites are considered the sample size is again small (n = 7) and the sites are assigned to three communities that somewhat correspond to the three original Full Network communities when sites from all time periods are considered (compare Figs 8 and 11). In the later period configuration the Northland site of Helena Bay (Site Community A in the full network) is grouped with Bream Head and Tamewhera (sites in the Full Network Site Community C). Pouerua Pa and Torpedo Bay are grouped together in the late period site network, whereas they were in separate communities in the full network. Notably, Mt. Wellington and Taputiketike Pa from the Full Network Site Community B, remain together. In this late period sampling, three source communities are defined instead of the two source communities of the original full network. These three source communities in the late period are formed by the bifurcation of the Full Network Source Community 1 into two separate groupings, with sources from the Full Network Source Community 2 being joined by the Cooks Beach source. Notably, the separation of the sites into early and late periods results in some site and source community coalescence and bifurcation but the distinction between the South-West Auckland sites of Mt Wellington and Taputiketike Pa (members of Full Network Site Community B) and all other sites is present.

## Discussion

Peeples’ [14] recent archaeological analysis of social networks in the American Southwest distinguishes between relational and categorical identification. These are two non-mutually exclusive modes of social identification that were created and maintained through differential processes. Relational identities were the result of networks of interpersonal interaction, created by direct and indirect connections between people. These identities did not involve explicit group membership or social roles, rather were often the result of interactions and exchanges of material culture. In contrast, categorical identification involved individuals explicitly identifying with others as members of larger groups. Membership in these groups could be consciously symbolized with material culture, with visually strong material being particularly affective. Peeples [14] suggests that dense relational networks of interaction were more susceptible to the creation of categorical identification, and in particular contexts the two could coalesce. The social network analysis of Aotearoa New Zealand obsidian artefacts provides insights into the relational and categorical connections of Māori identity.

Salmond [59] notes that Māori whakapapa (genealogy), and the social affiliations associated with these, can be conceptualized as complex networks, fluid and dynamic, that encompassed all forms of life. Whakapapa networks were the very fabric and key organizational principles of Māori society, permeating and defining fundamental social groupings [60]. Traditionally they were based on ambilineal descent qualified by residence and participation [60] with “…a tendency towards inclusivity rather than exclusivity” [61]. Ancestral networks were replicated and transported through interaction and exchanges, resulting in the maintenance of relationships and the creation of new social groups [59]. Some forms of Māori social affiliation, such as some hapū and iwi affiliations, could be considered categorical identifications as discussed by Peeples [14]. People within these groups actively identified through whakapapa with their relatives, and created and maintained rohe or tribal territories. While rohe were in some senses clearly defined, they were often fluid, existing as contended nonexclusive, overlapping areas of the landscape. Tangata whenua (people of a particular locality) in rohe accessed obsidian and created artefacts, and through this, created relational identities. Changes in the practices and spatial extent of accessing obsidian transformed both relational and categorical identities.

Archaeologists have begun to understand changing levels of Māori interaction and social affiliation. Walter et al. [62] suggest that during the colonization phase the viability of small communities was achieved through three processes: 1) colonization of the archipelago by an adequate number of people to create functioning groups with sufficient diversity for marriage partners of appropriate biological and social distance; 2) high levels of connectivity linking dispersed groups; and 3) one or more central places to serve as hubs in an extended communication network. Through systematic and coordinated exploration, an interaction network of viable communities throughout the archipelago was linked [62]. The evidence for this early network is primarily the distribution of lithic material. Mayor Island obsidian has been recovered from colonization phase sites in the far north and south of the archipelago, with argillites and basalts being widely dispersed. Anderson [5] [63] suggests that by AD1500 a growing sense of isolation had occurred, partially attributed to increasing population levels, a reduced sense of common ancestry, and greater social distance. With the development of territoriality, particularly in the optimal horticultural areas of northern Aotearoa New Zealand, there was a breakdown of long-distant interaction [30]. In these sites “the predominance of Mayor Island sources gave way quite quickly to increasing use of numerous wide-spread sources and then to local sources” and the distribution of argillites and basalts became much more restricted [63]. By the late pre-European contact period competition in some areas of Northland was intense, with fortified pa settlements providing protection and signalling control of territories. While there are indications that interaction was more limited there is some evidence of non-local lithic materials being accessed from far off locations [29] [30].

The results of the our SNA support the suggestions of previous researchers [21] [25] [26] [27] that Mayor Island obsidian played an important role in North Island sites. While Mayor Island was the dominant source material contained in the sites, material from other sources frequently occurred in site assemblages. It was the relative proportions of this other material that created similarity metrics of the individual sites, and these allowed the identification of site and source communities. Despite the large quantity of Mayor Island obsidian in the assemblages, that material did not significantly alter the community detection patterning, with three site communities and two source communities identified. The sites in Site Community A in Northland contained material from close sources, although there is some material from far off Mayor Island and Cooks Beach. The sites in Site Community B in South-West Auckland contained material from the far off sources of Te Ahumata, Whangamata, Rotorua, and Mayor Island, whereas the sites in Site Community C in North-East Auckland/Hauraki Gulf/Coromandel Peninsula were sourcing material from the Coromandel Peninsula and Bay of Plenty.

Previous researchers [30] [31] have proposed that the locations of sites influenced access to different obsidian sources, with “coastal” sites containing material from further sources and “inland” sites containing material from more local sources within smaller (30-50km) procurement catchments. While most of the sites in our analysis are relatively close to the coast, we note that both “coastal” and “inland” sites (e.g., Pouerua Pa and Taputiketike Pa) contained material from far-off sources. Least-cost distances were a factor in determining some assemblage compositions, particularly for the northern Site Community A sites. Sourcing material from relatively close sources could be a function of just minimizing energy expenditures, but could also reflect social and territorial constraints (c.f. [30]).

**Fig 12:**
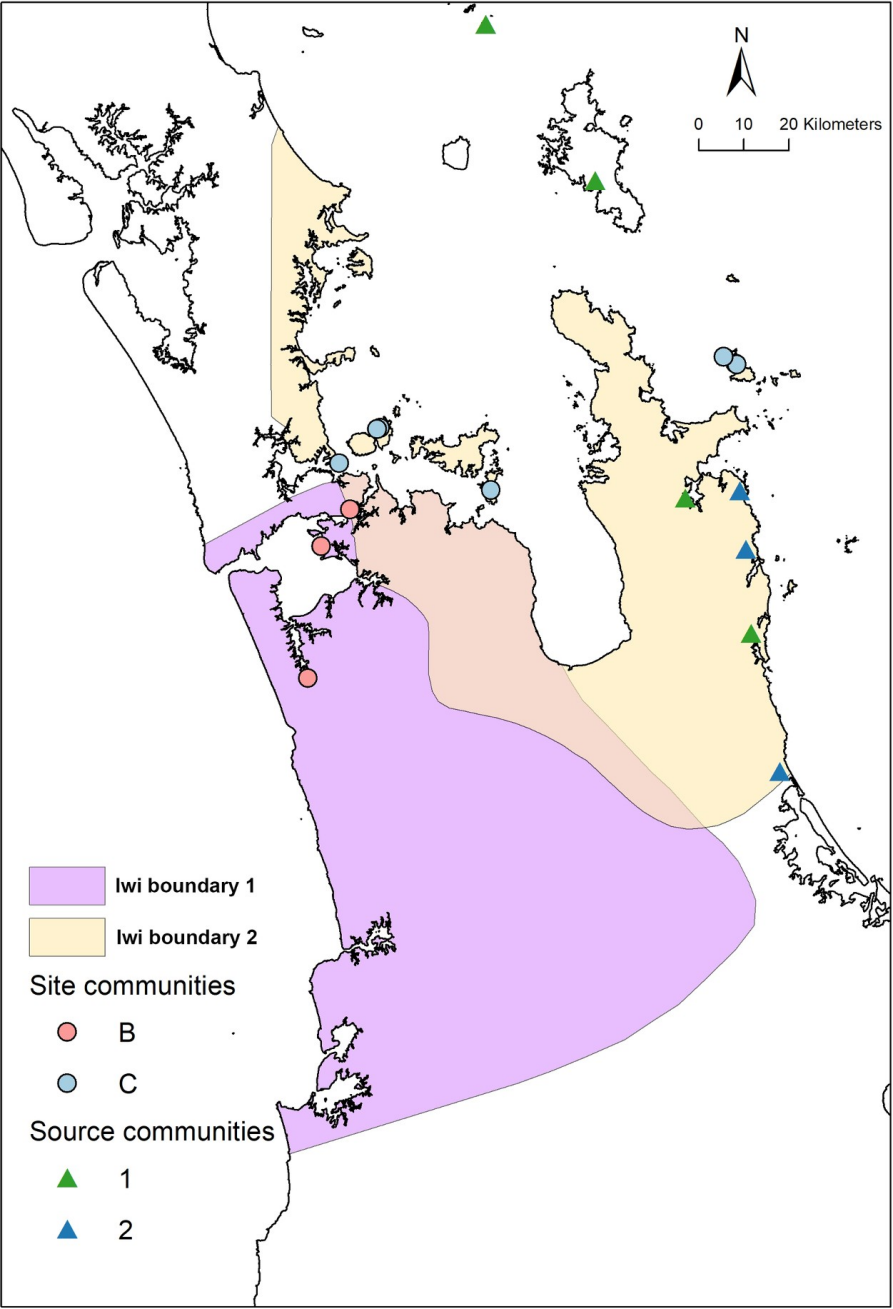
Examples of some contemporary iwi boundaries (http://www.tkm.govt.nz/) and the spatial distribution of obsidian source communities and archaeological site communities. Many other iwi have manawhenua authority in the region and have rohe territorial boundaries that overlap with those shown. The rohe territories shown were chosen to display the overlap in rohe and the coincidence between the boundaries and the source and archaeological site communities. Contains data sourced from the LINZ Data Service licensed for reuse under CC BY 4.0.

The South-West Auckland sites (Site Community C) and the North-East Auckland/Hauraki Gulf/Coromandel Peninsula sites (Site Community B) were influenced by social considerations in the procurement of material. Material in these sites came from select sources which were not necessarily the closest, and the spatially close sites in the South-West and North-East Auckland areas did not have similar assemblage compositions. The distinction in material culture between these two communities reflects differential relational identities of the occupants. These were created through patterned interactions with people living within and outside of these areas. The frequency of differentially sourced artefacts within site communities was likely a by-product of interactions and historical contingencies, and not the material manifestation of active symbolization of categorical identification. However, the role of social considerations in obsidian preferences (c.f. [29]) should not be overlooked. These relational identities, do however, coincide with some categorical identities associated with iwi. The spatial coincidence between the archaeologically documented division separating Site Community B and Site Community C and some contemporary iwi boundaries (http://www.tkm.govt.nz/) is marked (Fig 12). While we cannot conclusively associate the relational identity identified by the obsidian artefactual material and the categorical identity of current iwi, the coincidence is suggestive of a long established pattern and the possible coalescence of the two.

The social network analysis suggests that at a geographic scale of less than a distance of 110km people were within an interaction community that accessed similar proportions of obsidian from distinct sources. Within larger areas of distances of ca. 160km two communities were apparent, and within even larger areas of distances of 230km three or more communities likely existed. In Te Tai Tokerau, Tamaki, and Hauraki where our study area overlaps, the three site communities identified in this analysis are far fewer than the 36 iwi/hapū groups that have been formally recognised by the Crown (see http://www.tkm.govt.nz/). It is probable that increasing the sample of archaeological sites in our analysis would increase the number of identifiable site communities, but it is likely that the large geographic site communities that we have identified represent communities with relational identities that were spatially larger than known iwi and hapū. In some zones, however, where the development of relational identities was particularly strong and persistent it is likely categorically defined iwi and hapū developed with some boundaries that correspond to those identified by the social network analysis.

The temporal analysis of artefactual material is inconclusive due to small sample sizes, but it seems that the early sites formed fewer site communities and it was not until after AD1500 when additional sites are added to our sample that three communities are identified. These three communities form an analytical division that marks differences in assemblages between sites that are geographically quite close. While we cannot say for certain, this distinction is very likely to be the result of historical social interactions and categorical distinctions between the people who occupied these and other sites.

## Conclusion

Network analysis has identified discrete site and source communities on the basis of the composition of artefact assemblages and produced measures of the relative strengths of the relationships between them. The robust patterning of obsidian source frequency at archaeological sites does not directly correspond to geographical considerations. The source communities associated with these site communities are also not geographically close. Notably, while geographic distance influenced assemblage source compositions at some archaeological sites, divergence from the principles of acquiring material from the closest sources, or within small, local procurement areas, provides indications of social considerations. Sites within the Northland Site Community A were located close to each other and had similar compositions of artefact assemblages, as was the case for some sites within Site Community B and Site Community C. However, some sites within Site Communities B and C are located close to sites in the other community, yet contained very different compositions of source material in their artefact assemblages. This would suggest that people living in these relatively close locations had differential interaction levels, mobility patterns or procurement networks that were influenced by social considerations as opposed to purely least-cost geographic distances. While the temporal dimension of the spatial patterning is insecure due to small sample sizes, the community distinctions between sites that were geographically close was only identified when late period sites (post-AD 1500) were included in the analysis. Some of the site communities correspond to some boundaries of contemporary iwi rohe. We suggest that the relational identities associated with site community zones of interaction were significant in the development of iwi and hapū categorical identification. Future work on increasing sample sizes and refining chronological associations, possibly through the use obsidian hydration dating, will help clarify these and other aspects of Māori social affiliation.

## Supporting information

S1 Text A description of the revealed comparative abundance method. (DOCX)

S1 Table The artefacts and sources. (CSV)

S2 Table The frequency of artefacts from each source found at each site, and the least-cost travel times (hours) between archaeological sites and obsidian sources (A) the cost of travel over the ocean being the same as travel over flat land; and B) the cost of travel over the ocean being double the cost of travel over flat land.
(CSV)

S3 Table The mass-frequency self-similarity values for archaeological sites. (CSV)

S1 Fig A bipartite network of obsidian sources and archaeological sites, with links (edges) indicating the mass of artefacts associated with each source-site pair. (TIF)

S2 Fig The least-cost paths between Pouerua Pa and all obsidian sources with the cost of travel over the ocean being the same as travel over flat land. (TIF)

S3 Fig The least-cost paths between Pouerua Pa and all obsidian sources with the cost of travel over the ocean being double the cost of travel over flat land. (TIF)

S4 Fig The number of node changes in site communities when additional, randomly assigned artefacts are introduced to the network. (TIF)

S5 Fig The likelihood of each archaeological site changing site communities when additional, randomly assigned artefacts are introduced to the network. (TIF)

## References

[1] Jacomb C Holdaway R, Allentoft M, Bunce M, Oskam C, Walter R. High-precision dating and ancient DNA profiling of moa Aves: Dinornithiformes eggshell documents a complex feature at Wairau Bar and refines the chronology of New Zealand settlement by Polynesians. Journal of Archaeological Science, vol. 50, p. 24–30, 2014.

[2] Walter R, Smith I, Jacomb C. Sedentism, subsistence and socio-political organization in prehistoric New Zealand. World Archaeology, vol. 38, no. 2, pp. 274–290, 2006.

[3] Anderson A, Smith I. The transient village in southern New Zealand. World Archaeology, vol. 27, pp. 359–371, 1996.

[4] Walter R, Jacomb C, Bowron-Muth S. Colonisation, mobility and exchange in New Zealand prehistory. Antiquity, vol. 84, p. 497–513, 2010.

[5] Anderson A, Binney J, Harris A. Tangata Whenua: An Illustrated History, Wellington: Bridget Willams Books, 2014.

[6] Horde Allen A. and Hapu. in Oceanic Culture History: Essays in Honour of Roger Green, New Zealand Journal of Archaeology Special Publication, 1996.

[7] Iwi Ballara A., Wellington: Victoria University Press, 1998.

[8] Sissons J, Hongi W, Hohepa P. The Pūriri Trees are Laughing: A Political History of Ngā Puhi in the Inland Bay of Islands, Penguin, 1987.

[9] Davidson J. The Prehistory of New Zealand, Longman Paul, 1984.

[10] Sheppard P. Moving stones: comments on the archaeology of spatial interaction in New Zealand. in Change through Time, 50 Years of New Zealand Archaeology, NZAA Monograph 26, 2004.

[11] Earle T. Exchange systems in prehistory. in Trade and Exchange: Archaeological Studies from History and Prehistory, Springer, 2010.

[12] Freund K. An assessment of the current applications and future directions of obsidian sourcing studies in archaeological research. Archaeometry, vol. 55, no. 5, p. 779–793, 2013.

[13] Mol A, Hoogland M, Hofman C. Remotely local: Ego-networks of Late Pre-colonial AD 1000–1450 Saba, North-eastern Caribbean. Journal of Archaeological Method Theory, vol. 22, p. 275–305, 2015.

[14] Peeples M. Connected Communities: Networks, Identity, and Social Change in the Ancient Cibola World, The University of Arizona Press, 2018.

[15] Shennan M. Archaeological Approaches to Cultural Identity, Unwin Hyman, 1989.

[16] Baur A, Agbe-Davis A. Trade and interaction in archaeology. in Network Analysis in Archaeology: New Approaches to Regional Interaction, Oxford, Oxford University Press, 2013.

[17] Stein G. From passive periphery to active agents: emerging perspectives in the archaeology of interregional interaction. American Anthropologist, vol. 104, no. 3, pp. 903–916, 2002.

[18] IHodder I. Entangled: An Archaeology of the Relationships Between Humans and Things, John Wiley & Sons, 2012.

[19] Gjesfjeld E. Nework analysis of archaeological data from hunter-gatheres: methodological problems and potential solutions. Journal of Archaeological Method and Theogy, vol. 22, pp. 182–205, 2015.

[20] Borck L, Mills B, Peeples M, Clark J. Are Social Networks Survival Networks? An Example from the Late Pre-Hispanic US Southwest. Journal of Archaeological Method and Theory, vol. 22, pp. 33–57, 2015.

[21] Sheppard P, Irwin G, Lin S, McCaffrey C. Characterisation of New Zealand Obsidian using PXRF. Journal of Archaeological Science, vol. 38, no. 1, pp. 45–56., 2011.

[22] Leach BF, Four centuries of community interaction and trade in Cook Strait, New Zealand. Mankind, vol. 11, no. 3, pp. 391–405, 1978.

[23] Fredericksen CFK, Sewell B. The Reliability of Flaked Tool Function Studies in New Zealand Archaeology. Archaeology in Oceania, vol. 26, no. 3, pp. 123–126., 1991.

[24] Morwood M. A functional analysis of obsidian flakes from three archaeological sites on Great Barrier Island and one at Tokoroa. Records of the Auckland Institute and Museum, pp. 77–99, 1974.

[25] Cruickshank A. A Qualitative and Quantitative Analysis of the Obsidian Sources on Aotea Great Barrier Island and their archaeological significance, MA Thesis. University of Auckland, 2011.

[26] Kneebone B. Spatial Interactions and Communications: A Geochemical Analysis of Obsidian from the Tamaki region, BA Hons Dissertation, University of Auckland, 2016.

[27] Phillipps R, McAlister A, Allen M. Occupation duration and mobility in New Zealand prehistory: Insights from geochemical and technological analyses of an early Maori stone artefact assemblage. Journal of Anthropological Archaeology, vol. 42, pp. 105–121, 2016.

[28] Moore P, Coster J. Evidence of a well-developed obsidian distribution network in the far north of New Zealand. Journal of Pacific Archaeology, vol. 6, no. 1, pp. 1–17, 2015.

[29] McCoy MD, Carpenter J. Strategies for obtaining obsidian in pre-European contact era New Zealand. PLoS One, vol. 9, no. 1, p. e84302, 2014 10.1371/journal.pone.0084302 24416213 PMC3885548

[30] McCoy MD, Ladefoged TN, Codlin M, Sutton D. Does Carneiro’s circumscription theory help us understand Maori history? An analysis of the obsidian assemblage from Pouerua Pa, New Zealand Aotearoa. Journal of Archaeological Science, vol. 42, pp. 467–475, 2014.

[31] McCoy MD, Ladefoged TN, Blanshard A, Jorgensen A. Reconstructing Lithic Supply Zones and Procurement Areas: An Example from the Bay of Islands, Northland, New Zealand. Journal of Pacific Archaeology, vol. 1, no. 2, pp. 174–183., 2010.

[32] Birch J, Hart J. Social Networks and Northern Iroquoian Confederacy Dynamics. American Antiquity, vol. 83, no. 1, pp. 13–33, 2018.

[33] Collar A, Coward F, Brughmans T, Mills B. Networks in Archaeology: Phenomena, Abstraction, Representation. Journal of Archaeological Method and Theory, vol. 22, pp. 1–32, 2015.

[34] Evans T. Which network model should I use? Towards a quantitative comparison of spatial network models in archaeology. in The Connected Past: Challenges to Network Studies in Archaeology, Oxford, Oxford University Press, 2016.

[35] Knappett C. Network Analysis in Archaeology, Oxford: Oxford University Press, 2013.

[36] Mills B, Clark J, Peeples M, Haas W, Roberts J, Hill B, et al Transformation of social networks in the late pre-Hispanic US Southwest. Proceedings of the National Academy of Science, vol. 110, no. 15, pp. 5785–5790, 2013. 10.1073/pnas.1219966110 PMC3625298 23530201

[37] Ossa A. Using network expectations to identify multiple exchange systems. Journal of Anthropological Archaeology, vol. 32, p. 415–432., 2013.

[38] Pearce E, Moutsiou T. Using obsidian transfer distances to explore social network maintenance in late Pleistocene hunter–gatherers. Journal of Anthropological Archaeology, vol. 36, p. 12–20, 2014. 10.1016/j.jaa.2014.07.002 25214705 PMC4157217

[39] Freund K, Batist Z. Circulation and early maritime naviagation in the Neolithic as shown through social network analysis. Journal of Island and Coastal Archaeology, vol. 9, pp. 364–380, 2014.

[40] Ibanez JJ, Ortega D, Campos D, Khalidi L, Mendez V. Testing Complex Networks of Interaction at the Onset of the Near Eastern Neolithic Using Modelling of Obsidian Exchange. Journal of the Royal Society Interface, vol. 12, no. 107, p. 10.1038/309, 2015. PMC4590507 25948614

[41] Phillips S, Gjesfjeld E. Evaluating adaptive network strategies with geochemical sourcing data: A case study from the Kuril Islands. in Network Analysis in Archaeology: New Approaches to Regional Interaction, Oxford University Press, 2013.

[42] Golitko M, Meierhoff J, Feinman G, Williams P. Complexities of collapse: the evidence of Maya obsidian as revealed by social network graphical analysis. Antiquity, vol. 86, p. 507–523., 2012.

[43] McCoy MD, Jorgensen A, Glover H, Stevenson C, Kneebone B, Cruickshank A, et al Geochemical Sourcing of New Zealand Obsidians by Portable X-Ray Florescence from 2011 to 2018. Journal of Open Archaeology Data, 10.5334/joad.52. 2019.

[44] McCoy MD, Jorgensen A, Glover H, Stevenson C, Kneebone B, Cruickshank A, et al Data for Geochemical Sourcing of New Zealand Obsidians by Portable X-Ray Fluorescence from 2011 to 2018. 2019.

[45] Holdaway SJ, Stern N, Chauhan P. A Record in Stone: The Study of Australia's Flaked Stone Artefacts, Canberra: Aboriginal Studies Press, 2004.

[46] Fanning P, Holdaway SJ. Artifact visibility at open sites in western New South Wales, Australia. Journal of Field Archaeology, vol. 29, no. 3–4, pp. 255–271, 2004.

[47] Douglass M. The archaeological potential of informal lithic technologies: a case study of assemblage variability in western New South Wales, Australia, Unpublished Ph.D Thesis, University of Auckland, ResearchSpace@ Auckland, 2010.

[48] Holdaway SJ, Douglass M. A Twenty-First Century Archaeology of Stone Artifacts. Journal of Archaeological Method and Theory, vol. 19, no. 1, pp. 101–131, 2012.

[49] Schick K. Experimentally-derived criteria for assessing hydrologic disturbance of archaeological sites. in Natural formation processes and the archaeological record, BAR International Series 352, 1987, pp. 86–107.

[50] Fanning P, Holdaway SJ. Stone artifact scatters in western NSW, Australia: Geomorphic controls on artifact size and distribution. Geoarchaeology, vol. 16, no. 6, pp. 667–686, 2001.

[51] Lothrop J, Burke A, Winchell-Sweeney AGG. Coupling lithic sourcing with least cost path analysis to model Paleoindian pathways in Northeastern North America. American Antiquity, vol. 83, no. 3, pp. 462–484, 2018.

[52] Supernant K. Modeling Métis mobility? Evaluating least cost paths and indigenous landscapes in the Canadian West. Journal of Archaeological Science, vol. 84, pp. 63–73, 2017.

[53] Gustas R, Supernant K. Least cost path analysis of early maritime movement on the Pacific Northwest Coast. Journal of Archaeological Science, vol. 78, pp. 40–56, 2017.

[54] Tobler W. Three Presentations on Geographical Analysis and Modeling, National Center For Geographic Information and Analysis Technical Report 93–1. University of California Santa Barbara., 1993.

[55] Blondel V, Guillaume J, Lambiotte R, Lefebvre E. Fast unfolding of communities in large networks. Journal of Statistical Mechanics: Theory and Experiment, vol. 10, p. 10008, 2008.

[56] Balassa B. Trade Liberalisation and ‘Revealed’ Comparative Advantage. The Manchester School, vol. 33, no. 2, p. 99–123, 1965.

[57] Balassa B, Noland M. Revealed Comparative Advantage in Japan and the United States. Journal of International Economic Integration, vol. 4, no. 2, pp. 8–22, 1989.

[58] Hidalgo C, Klinger B, Barabasi A, Hausmann R. The Product Space Conditions the Development of Nations. Science, vol. 317, no. 5837, p. 482–87, 2007 10.1126/science.1144581 17656717

[59] Salmond A. Tears of Rangi: Experiments Across Worlds, Auckland: Auckland University Press, 2017.

[60] Kawharu M, Newman E. Whakapaparanga: Social Structure, Leadership and Whāngai. in Te Kōparapara: An Introduction to the Māori World, Auckland, University of Auckland Press, 2018.

[61] Sutton D. Organisation and ontology: The orgins of the northern Maori chiefdom, New Zealand. Man, vol. 25, no. 4, pp. 667–692, 1990.

[62] Walter R, Buckley H, Jacomb C, Matisoo-Smith E. Mass Migration and the Polynesian Settlement of New Zealand. Journal of World Prehistory, vol. 30, no. 4, pp. 351–376, 2017.

[63] Anderson A. The prehistory of South Polynesia. in The Oxford Handbook of Prehistoric Oceania, New Yor, Oxford University Press, 2018.

