# Correction: Social network analysis of obsidian artefacts and Māori interaction in northern Aotearoa New Zealand

**DOI:** 10.1371/journal.pone.0216420

**Published:** 2019-04-30

**Authors:** Thegn N. Ladefoged, Caleb Gemmell, Mark McCoy, Alex Jorgensen, Hayley Glover, Christopher Stevenson, Dion O’Neale

## Notice of Republication

An incorrect version of Fig 12 was published in error. This article was republished on April 19, 2019 to correct for this error. Please download this article again to view the correct version.
