# Supplementary material for: Social network analysis of obsidian artefacts and Māori interaction in northern Aotearoa New Zealand
Source: PLoS One. 2019 Mar 14;14(3):e0212941. doi: 10.1371/journal.pone.0212941 (PMC6417682; doi:10.1371/journal.pone.0212941)
Supplement: S1 Text — (DOCX) [file pone.0212941.s001.docx]

**Supplementary Text: 1**

**Revealed Comparative Abundance**

The size of assemblages from different study sites can vary greatly, as can the amount of material associated with different geological sources. In order to account for this, when determining the likelihood, we use the method of Revealed Comparative Abundance (RCA) which accounts for relative abundances of material from different sites and sources. The method is based on that used in economics where it is known as Revealed Comparative Advantage and is used, for example, to calculate the relative advantages that countries have in producing different exports, accounting for both the total exports from a country and the total world production of a certain class of exports. See, for example [56] [57] [58].

The definition of RCA is given by

$$RCA\left( s,g \right)= \frac{\frac{x(s,g)}{\sum_{g} x(s,g)}}{\frac{\sum_{s} x(s,g)}{\sum_{s,g} x(s,g)}}$$

where ***x(s,g)*** is the number of artefacts found at study site ***s*** which are associated with geological source ***g***. When RCA is greater than one, it indicates the share of artefacts at a study site, originating from a particular geological source is greater than the fraction of all artefacts from that source, relative to the total number of artefacts from all sources. That is site-source pairs with an RCA greater than one have more artefacts from that source than would be expected if artefacts from different sources were distributed uniformly at random across sites, but with the total number of artefacts at each site and from each source being preserved.
