# Supplementary figures and images for: Social network analysis of obsidian artefacts and Māori interaction in northern Aotearoa New Zealand
Source: PLoS One. 2019 Mar 14;14(3):e0212941. doi: 10.1371/journal.pone.0212941 (PMC6417682; doi:10.1371/journal.pone.0212941)

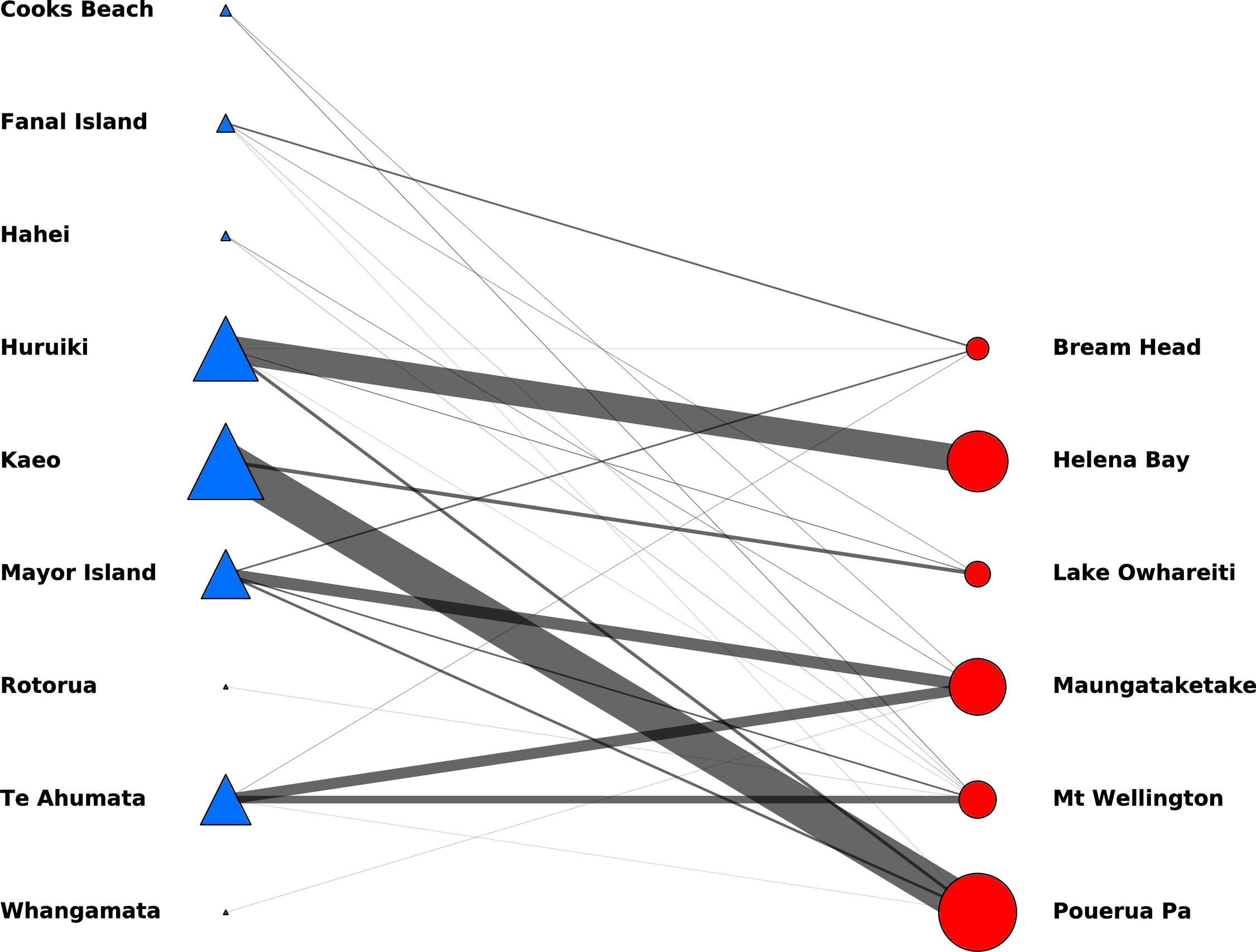

Supplement: S1 Fig — (TIF) [file pone.0212941.s005.tif]

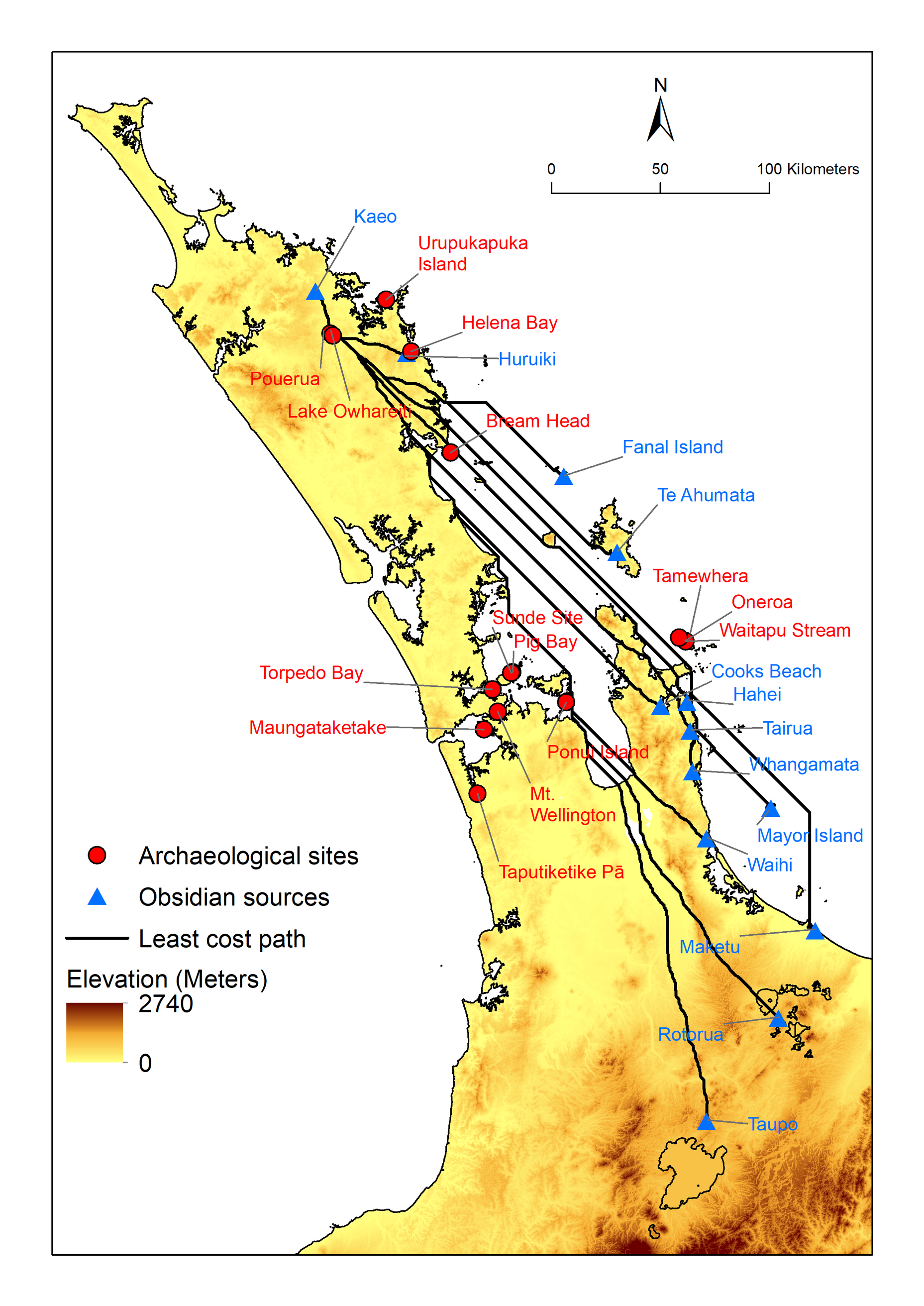

Supplement: S2 Fig — (TIF) [file pone.0212941.s006.tif]

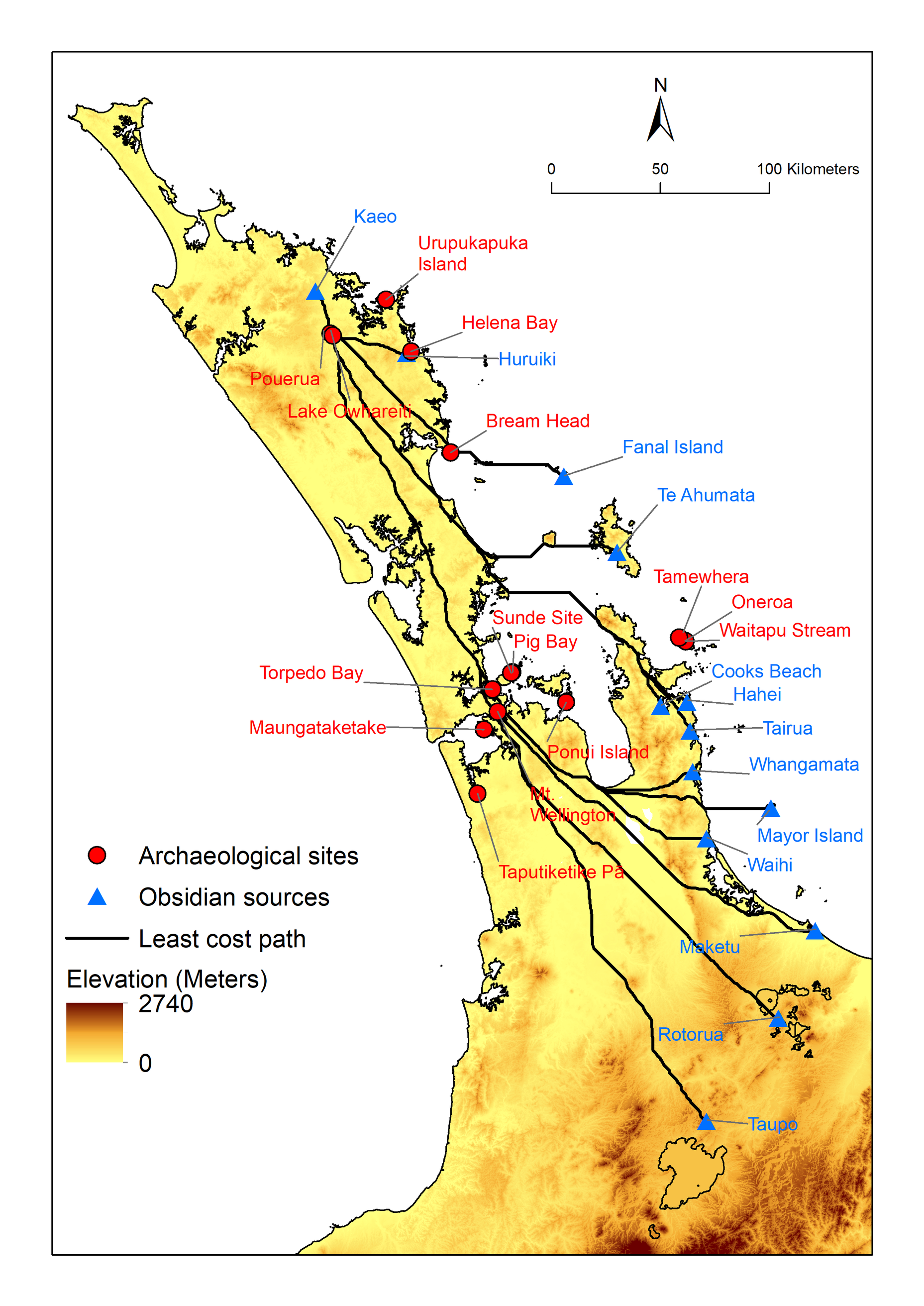

Supplement: S3 Fig — (TIF) [file pone.0212941.s007.tif]

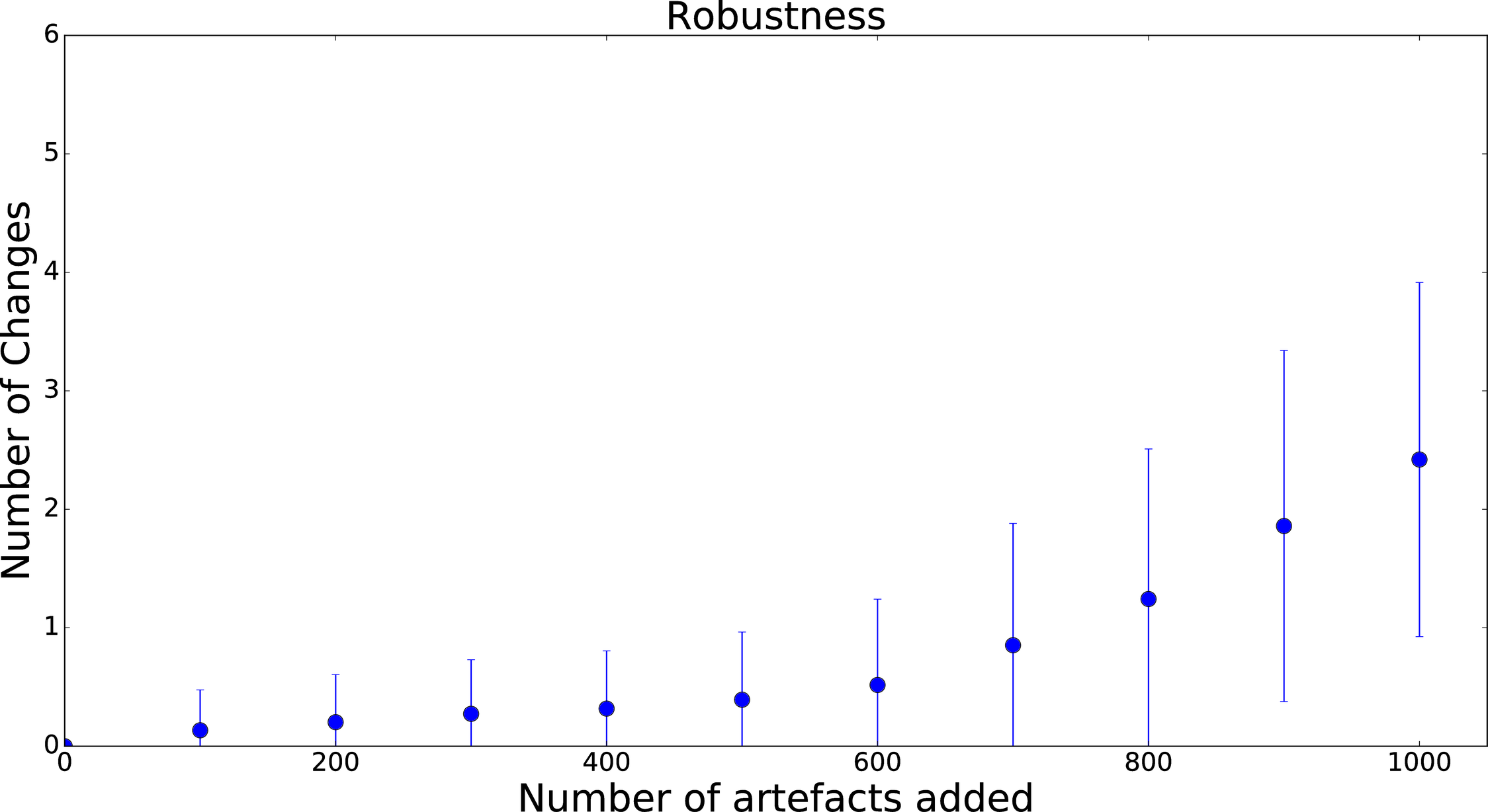

Supplement: S4 Fig — (TIF) [file pone.0212941.s008.tif]

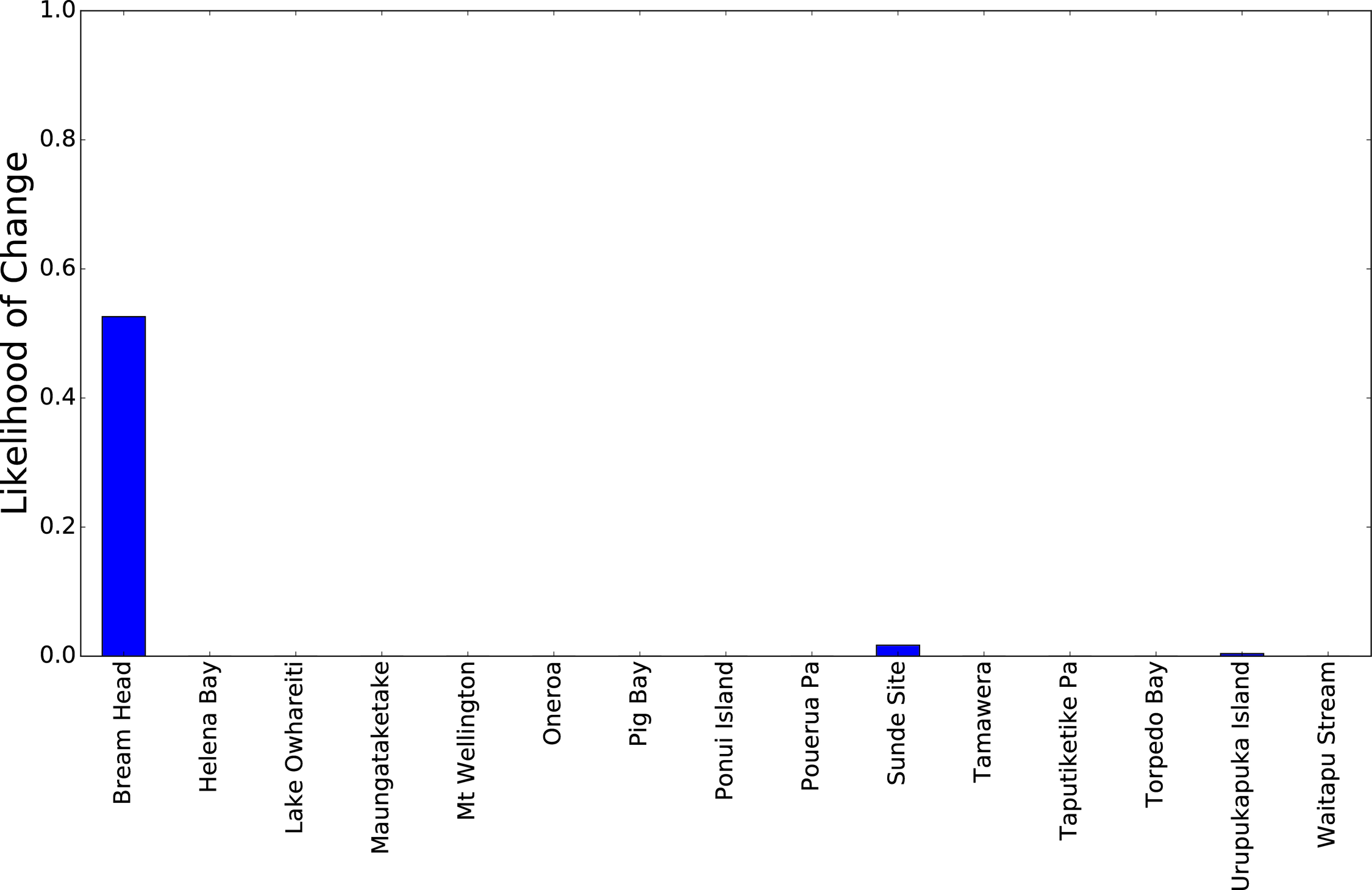

Supplement: S5 Fig — (TIF) [file pone.0212941.s009.tif]
